# Introduction to Mindfulness: Evidence-Based Medicine Lecture and Active Session

**DOI:** 10.15766/mep_2374-8265.10472

**Published:** 2016-09-28

**Authors:** Magdalena Pasarica, Ernestine Lee, Michael Lee

**Affiliations:** 1 Associate Professor, Department of Medical Education, University of Central Florida College of Medicine; 2 Associate Professor, Department of Medical Education, University of Texas at Austin Dell Medical School; 3 Assistant Professor, Department of Medical Education, University of Texas at Austin Dell Medical School

**Keywords:** Evidence-Based Medicine, Mindfulness, Practical Exercise

## Abstract

**Introduction:**

There is a growing body of evidence regarding the effectiveness of mindfulness practices and the benefits for the resilience of patients and medical professionals. Thus, there is a need to educate medical students in this technique and its benefits, but there are no readily available resources that emphasize the current evidence. We developed an introductory session for the mindfulness technique as an evidenced-based medicine lecture combined with an active mindfulness exercise.

**Methods:**

This session consisted of a PowerPoint lecture overview of mindfulness, a topic unknown to most students, with an emphasis on evidence-based facts, as well as an active practical mindfulness exercise. The effectiveness of the lecture was assessed using a survey instrument, which was designed to assess the learners' knowledge and interest in learning and using mindfulness (before and after the session) and their interest in integrating mindfulness into the medical school curriculum. The survey contained six multiple-choice questions. Participation was voluntary and anonymous, with the use of Turning Point technology (an audience response system).

**Results:**

The session was administered to and evaluated by first- and second-year medical students with no prior instruction in mindfulness. The session was held by a physician who uses mindfulness in the clinic. After the session, 90% of students were interested in learning more about mindfulness, and the majority were interested in having mindfulness integrated into the medical curriculum.

**Discussion:**

The results suggest the session can be effectively used as is in other medical teaching institutions to introduce students to mindfulness and to assess students' perception of knowledge in the area and their interest in both having more comprehensive training on the subject and practicing mindfulness in medical care.

## Educational Objectives

At the end of this session, learners will be able to:

1. Increase their perception of their own knowledge in mindfulness.
2. Increase their interest in using mindfulness to improve patient care and/or their own health.
3. Increase their interest in learning more about mindfulness.
4. Increase their interest in having mindfulness integrated into the medical curriculum.

## Introduction

Resilience is the ability to adapt to, respond to, and recover from current and potential life stressors. This can be achieved with mindfulness, which is defined as being aware of one's moment experience and feelings but concentrating on insight, relaxation, and putting aside worries and expectations. Rooted in a Buddhist tradition, mindfulness is a form of meditation whose aim is to alleviate pain and suffering. Although historically Buddhist, mindfulness meditation does not require any particular religious or cultural belief system.^1^ The pioneering work of Jon Kabat-Zinn and others has documented the benefits of mindfulness practice for many chronic health conditions, including chronic pain and depression.^2^ According to the Association of American Medical Colleges, “resident physicians are especially vulnerable and their well-being must be accorded the highest priority … [g]iven the uncommon stresses inherent in fulfilling the demands of their training program.”^3^ Recent work has illustrated the benefits of mindfulness practices for the well-being of medical students and residents.^4–6^ Assessment questions on mindfulness have already been integrated into the medical board exams. Fourteen medical schools have mindfulness integrated into their curriculum already,^4^ some schools have elective courses,^7^ and interest in mindfulness is growing. However, resources for mindfulness education are sparse.

As the body of evidence for mindfulness effectiveness grows and the technique begins to be used in traditional medical practice, instruction on this technique and its benefits is needed at the medical school level. To our knowledge, there are no readily available resources that introduce students to the health benefits of mindfulness. As with any technique that has been used for a long time but only recently proven to be scientifically sound, there is a perceived hesitancy of usage amongst some medical providers and educators. The need for this session was prompted by discussions with our first-year medical students during a session on how to properly deal with powerful emotions as a physician. Some students knew a little about the technique and inquired about the science behind it, while some students knew nothing about it. However, all students expressed interest in learning more.

We recognized that there was a lack of mindfulness training in our medical curriculum. We hypothesized that exposing our medical school students to the scientific evidence on both the technique and benefits of mindfulness and giving them an opportunity to undergo a mindfulness exercise would (1) increase their interest in learning more about mindfulness and in using mindfulness to improve patient care and their own health and (2) generate data showing their interest in having mindfulness integrated into the curriculum, which could be presented in future discussions regarding the potential inclusion of mindfulness education in our medical curriculum.

Thus, we developed a session that can be used to introduce students to mindfulness and the evidence supporting its efficiency in medical practice. We are experienced medical educators with extensive knowledge in mindfulness, so we used our own expertise. The main author successfully uses mindfulness in her clinical practice. By the end of the session, we expect students to become familiar with the technique and the evidence supporting its use in medical training and to be stimulated to read more and/or take formal courses to advance their mindfulness skills. Our long-term goal is to give students a tool that can be used to improve their own coping with stress, chronic illness, pain, and life trauma and that may also be applicable to their patients to help them develop positive coping skills as an alternative to the unconscious development of psychosomatic symptoms.

Participant students were surveyed before and after the session to determine if they became interested in learning more about mindfulness and if they would like to have mindfulness integrated into their curriculum. The purpose of the survey was to both prove the efficacy of the session and gather data to support a potential discussion on developing a mindfulness course at our school.

This session was by no means intended to fully educate students in mindfulness; it was meant to prime their interest with evidence and data while demonstrating a need for more curricula around this area. This session was implemented at the University of Central Florida College of Medicine as part of elective presentations organized by our Family Medicine Interest Group (which is advised by the first author). Any student enrolled at our school was eligible for participation. The session was designed to have two components: an evidence-based medicine lecture and an active component. We elected to use an active component of experiencing a mindfulness exercise as we know that students respond better and are more engaged in active learning. Our data suggest that this session accomplished our goals and could be applied to other schools and therefore would be useful for the medical education community. This session could be used in any school where mindfulness is not part of the curriculum or in schools where mindfulness is part of the elective curriculum.

## Methods

This session is original and can be implemented as is by other instructors of medical professionals. This session was implemented with the medical students at the University of Central Florida College of Medicine, who also evaluated the session as described in the Results section. The session sets out to (1) define mindfulness, its framework, and the evidence of mindfulness in medicine; (2) describe the potential benefits of mindfulness techniques for both medical care and self-resilience; and (3) provide the experience of a mindfulness exercise.

This session consisted of a lecture, as well as an active practical mindfulness exercise (Appendix A). The PowerPoint lecture was designed to give an overview of mindfulness, a topic unknown to most students, with an emphasis on evidence-based facts. We opted to add an active exercise of mindfulness guided by the presenter so that learners can experience themselves how easy and effective it is to use mindfulness techniques in their day-to-day life. The session has three components: (1) a discussion of the definition of mindfulness and its framework; (2) a discussion of evidence supporting the effectiveness of mindfulness in practice, including a summary of relevant published work; and (3) a link to actively experience a mindfulness exercise where the instructor provides the framework and experiences the 3-minute exercise with the learners.

Learner engagement during the session was assessed by the expert opinion of the instructor. The effectiveness of the lecture was assessed using a survey instrument (Appendices B & C), which was designed to assess the learners' knowledge and interest in learning and using mindfulness (before and after the session) and their interest in integrating mindfulness into the medical school curriculum. The survey contained six multiple-choice questions. Participation was voluntary and anonymous, with the use of Turning Point technology (an audience response system). This technology can be used to assess the efficiency of the session and the interest your students have in learning more about mindfulness and having it as formal training in their curriculum. This survey can be further used as supporting evidence for instructors who want to integrate mindfulness in their school curriculum. The survey is presented in two forms: a PowerPoint/Turning Point version that can only be used by instructors who have access to the Turning Point technology (Appendix B) and a paper/pencil version for instructors who do not have access to that technology (Appendix C). Please note that if there is no access to the Turning Point technology, the PowerPoint version of Appendix B will still be visualized, although the Turning Point function will not work.

The audience was composed of first- and second-year medical students. The session was held during lunch (60 minutes) in May of 2015, and it was part of the Family Medicine Interest Group sessions, but not part of the curriculum. It was led by one experienced instructor who has practiced mindfulness in the clinic for approximately 2 years. Forty-four students participated, and attendance was voluntary. For this activity, a computer with PowerPoint capabilities, internet access, and Turning Point technology was used. The study was approved by the Institutional Review Board at the University of Central Florida.

## Results

We used a survey containing six multiple-choice questions to assess learners' knowledge of and interest in mindfulness. A majority of students answered the survey (*N* = 29–38 responses), as indicated in the Table.

**Table.** Mindfulness Assessment Survey Questions and Results (*N* = 29–38)

Question and Response	Pretest (%)	Posttest (%)
How much do you know about mindfulness at this time?		
A lot/enough to use in clinical practice	3	29
Moderate	22	53
A little	32	13
Nothing	33	5
Are you interested in using mindfulness to improve patient care and/or your own health?		
Yes	71	87
Not sure at this time	29	10
No	0	3
Are you interested in learning more about mindfulness?^a^		
Yes		90
Not sure at this time		7
No		3
Would you like to have mindfulness integrated in your medical school curriculum?^a^		
Yes		68
Not sure at this time		16
No		16

a Question not asked on pretest.

We first asked a question to assess learners' mindfulness knowledge before and after the session. Our results show that before the session, 3% of the students knew enough about mindfulness to use it in practice, and 33% reported that they knew nothing about it. The majority knew a moderate amount (22%) or little (42%). After the session, 29% of the students reported they knew enough about mindfulness to use it in practice, and only 5% reported that they knew nothing about it. The majority of learners reported they knew a moderate amount (53%) or little (13%).

We then asked a question assessing learners' interest in using mindfulness in clinical practice and/or towards their own health before and after the session. Our results show that before the session, 71% of the students reported they were interested in using mindfulness, 29% were not sure at that time, and none reported that they were not interested in using mindfulness. After the session, 87% of the students reported they were interested in using mindfulness, and 10% were not sure. Only 3% of the students reported that they were not interested in using mindfulness.

After the session, learners were asked about their interest in learning more about mindfulness and if they wanted to have mindfulness integrated into their curriculum. Our results show that 90% of the students reported they were interested in learning more about mindfulness, and 7% were not sure. Only 3% of the students reported that they were not interested in learning more. An overwhelming 68% of the students were interested in having mindfulness integrated into their curriculum, and 16% were not sure. Only 16% of the students reported that they were not interested in having mindfulness integrated into their curriculum.

Students appeared engaged during the session, and they all participated in the active part. Multiple students expressed their interest in mindfulness and asked for more resources.

## Discussion

There is a growing body of knowledge regarding mindfulness practices and their benefits for patients and medical trainees. Our results show that in just one 60-minute session of evidence-based medicine background and a brief mindfulness experience, 90% of students were interested in learning more about mindfulness practice. In addition, the majority of students expressed their interest in having mindfulness integrated into their medical curriculum. It is important to note that this was a nonrequired session, and therefore, it may have brought together a group of students who were either interested in mindfulness or were open to such a technique. A possible challenge one may encounter in a required session is the participation of students who may not be open to this new technique. However, this comprehensive session should at least pique a student's interest and drive him or her to seek out more about this technique. Another limitation of this study is that we cannot identify whether students who had moderate or little knowledge about mindfulness stayed in the same category after the intervention. Most likely, the groups got redistributed in the final postintervention categories (e.g., the learners who knew nothing may have moved to the groups who learned a small/little amount, a moderate amount, or enough to use in clinical practice, and the learners who knew little moved into the moderate and/or enough-to-use-in-clinical-practice groups).

Given the positive results, we believe this session can be implemented at other institutions where mindfulness is not part of the curriculum to introduce students to mindfulness and to assess their interest in having more comprehensive training on the subject. We suggest that participation in this session be voluntary, with no prerequisites needed. This can serve as an introduction to the topic of mindfulness. A room large enough to accommodate all students comfortably is needed. We recommend an instructor who is using mindfulness in his or her clinical practice or is knowledgeable in this area. The instructor should be able to answer practical questions during and after the session. The instructor should share his or her own experience using mindfulness. We believe that the selection of an experienced instructor is very important for the success of the session. A computer with PowerPoint presentation capabilities and internet access is needed. In addition, if the survey is deployed, then we recommend using either an audience response system technology (if available) to capture students' answers or a paper survey (both options are attached in Appendices B and C, respectively, and can be used without modification).

This readily available, effective resource will advance the integration of mindfulness in medical training, increasing medical practitioner knowledge and interest in using mindfulness, with the ultimate goal of improving patient and physician resilience. Future studies can continue to identify the optimal time to include mindfulness and other self-care modalities in the medical training continuum. Future research may also identify trainees who might optimally benefit based on previous life experiences or openness to the practice of mindfulness as a method of stress reduction and improving resiliency. In addition to the presented introductory session, more readily available resources are needed for instructors who are planning to introduce a curriculum on advanced mindfulness training for medical professionals. Adding a longitudinal course in medical education and/or in residency may increase the likelihood of learners using this technique and could be an interesting future study; however, that is beyond the scope of this session.

## Appendices

A. Mindfulness Presentation.pptx
B. Survey-Electronic.pptx
C. Survey-Paper.docx All appendices are peer reviewed as integral parts of the Original Publication.
