# Supplementary material for: Introduction to Mindfulness: Evidence-Based Medicine Lecture and Active Session
Source: MedEdPORTAL. 2016 Sep 28;12:10472. doi: 10.15766/mep_2374-8265.10472 (PMC6464426; doi:10.15766/mep_2374-8265.10472)
Supplement: Supplementary file 1 — A. Mindfulness Presentation.pptx B. Survey-Electronic.pptx C. Survey-Paper.docx [file mep-12-10472-s001.zip › A. Mindfulness Presentation.pptx]

## Slide 1
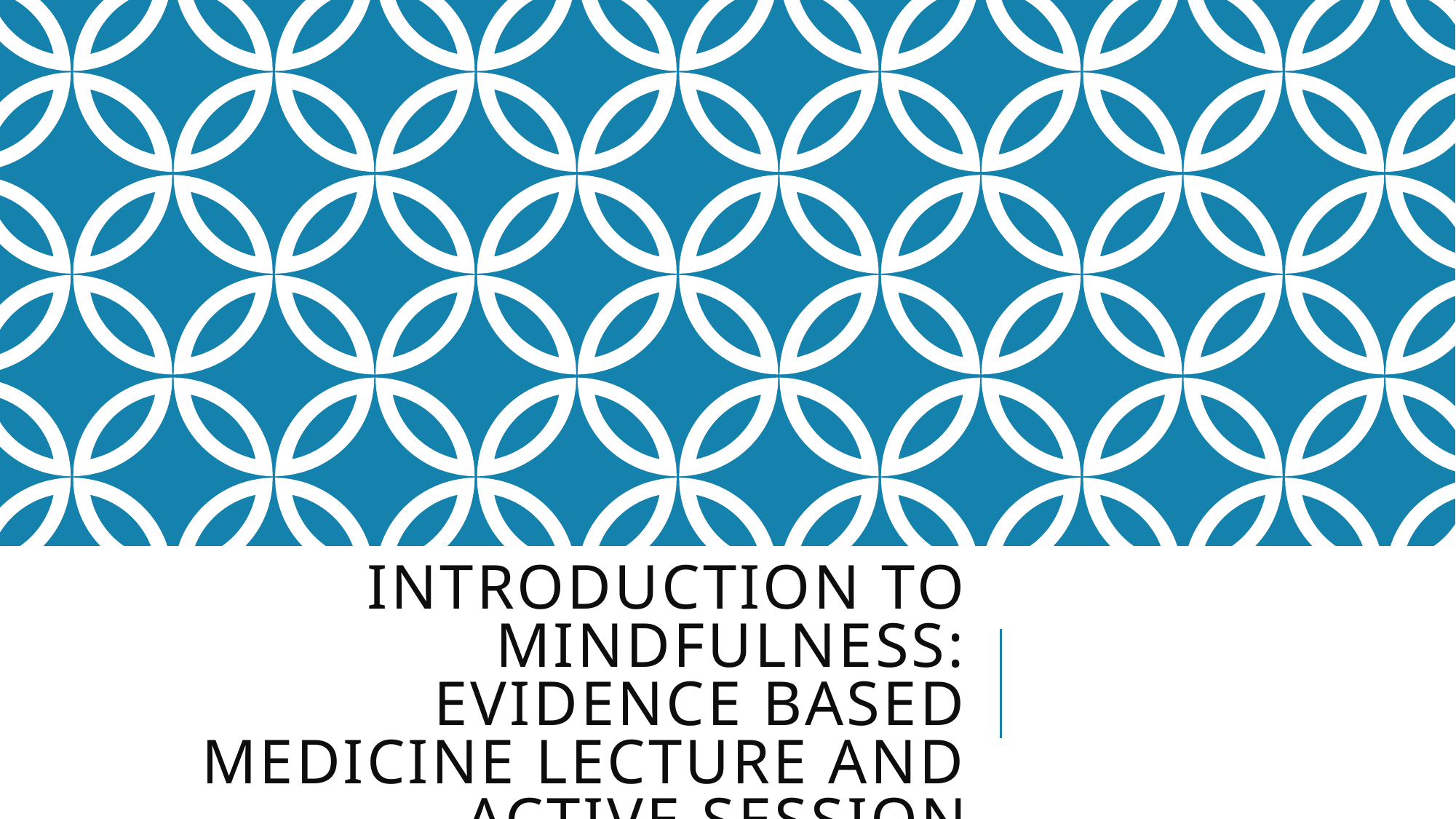

# Introduction to mindfulness: evidence based medicine lecture and active session

## Slide 2
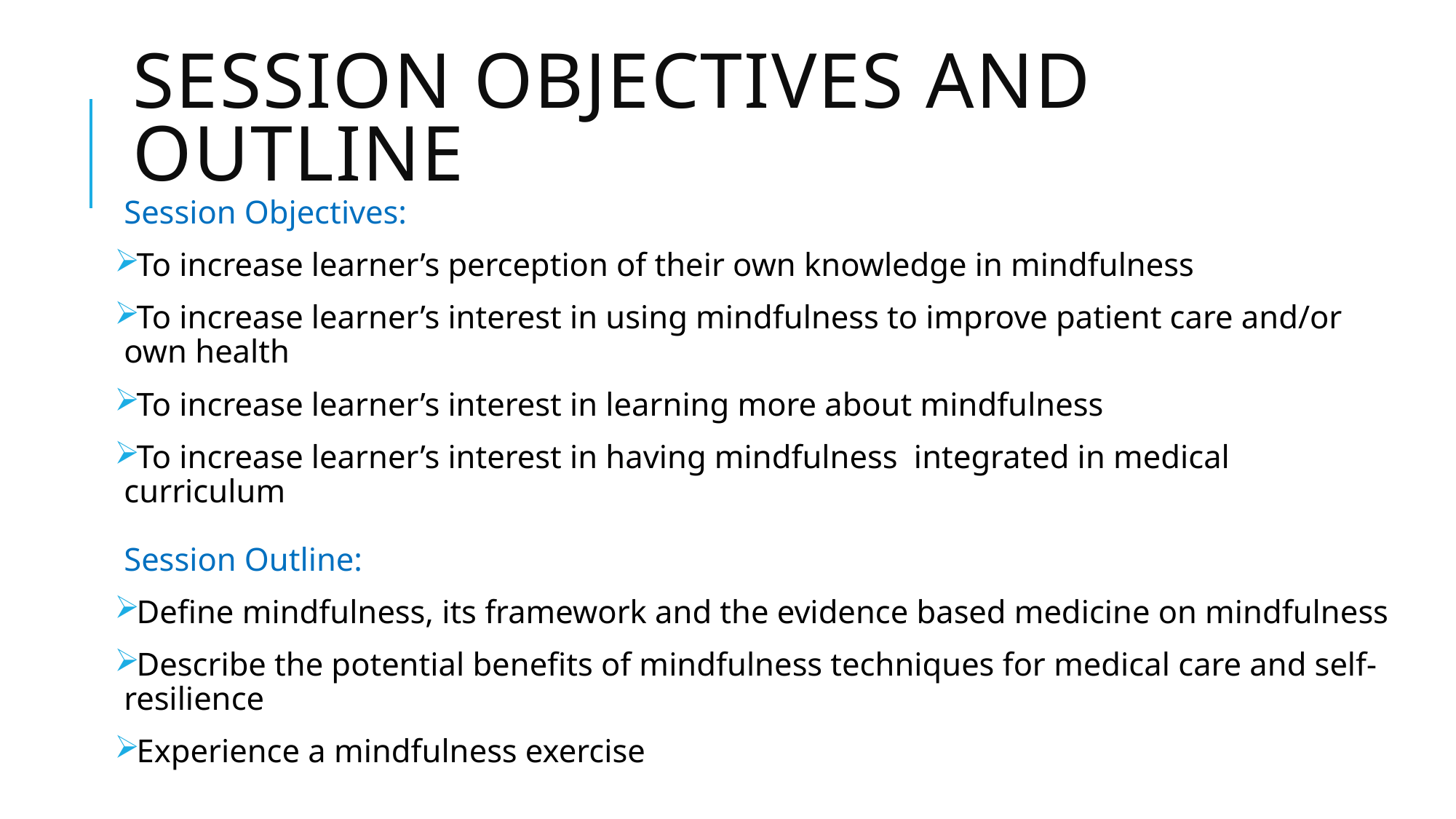

# Session objectives and outline
Session Objectives:
To increase learner’s perception of their own knowledge in mindfulness
To increase learner’s interest in using mindfulness to improve patient care and/or own health
To increase learner’s interest in learning more about mindfulness
To increase learner’s interest in having mindfulness integrated in medical curriculum
Session Outline:
Define mindfulness, its framework and the evidence based medicine on mindfulness
Describe the potential benefits of mindfulness techniques for medical care and self-resilience
Experience a mindfulness exercise

## Slide 3
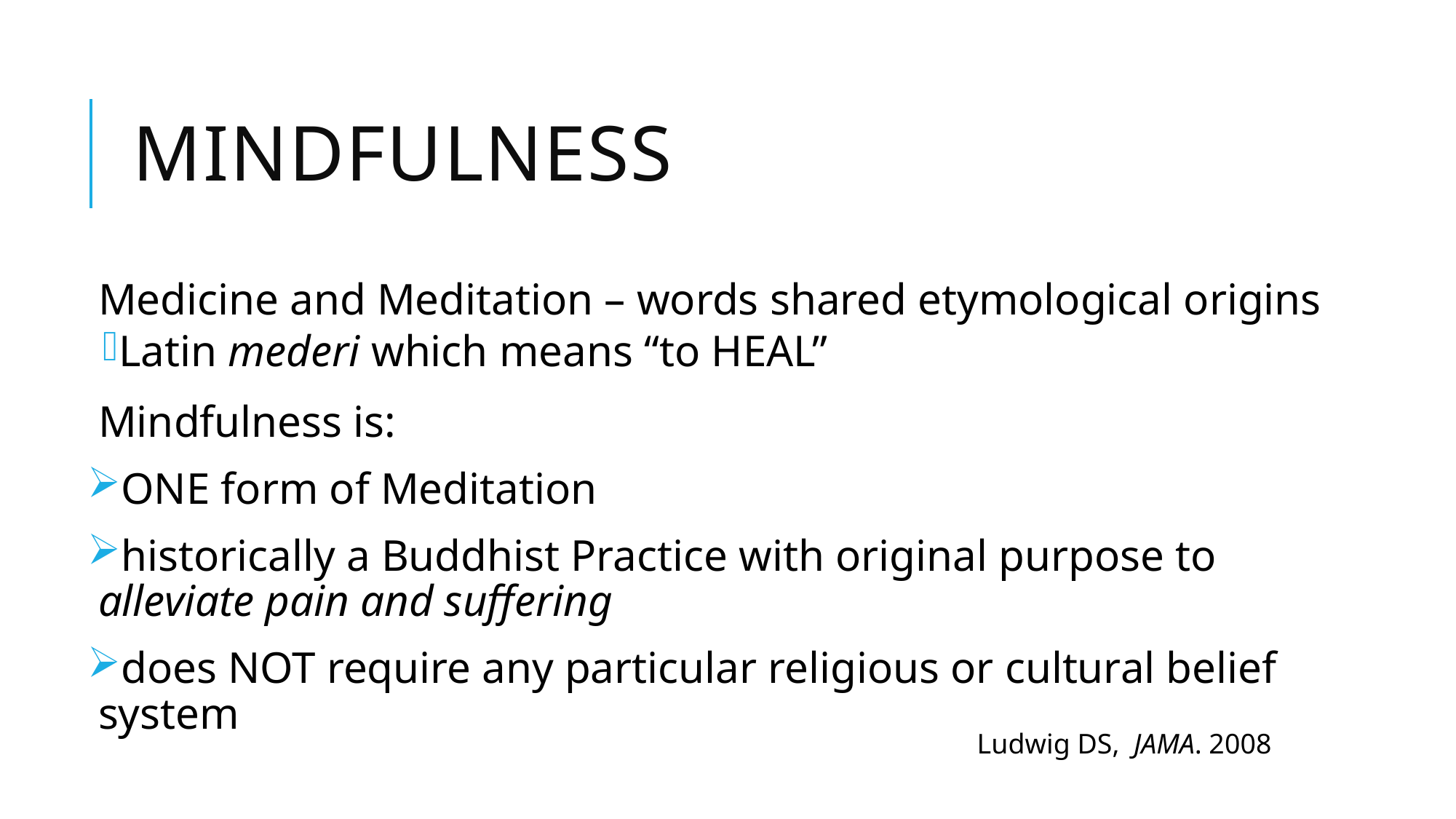

# Mindfulness
Medicine and Meditation – words shared etymological origins
Latin mederi which means “to HEAL”
Mindfulness is:
ONE form of Meditation
historically a Buddhist Practice with original purpose to alleviate pain and suffering
does NOT require any particular religious or cultural belief system
Ludwig DS, JAMA. 2008

## Slide 4
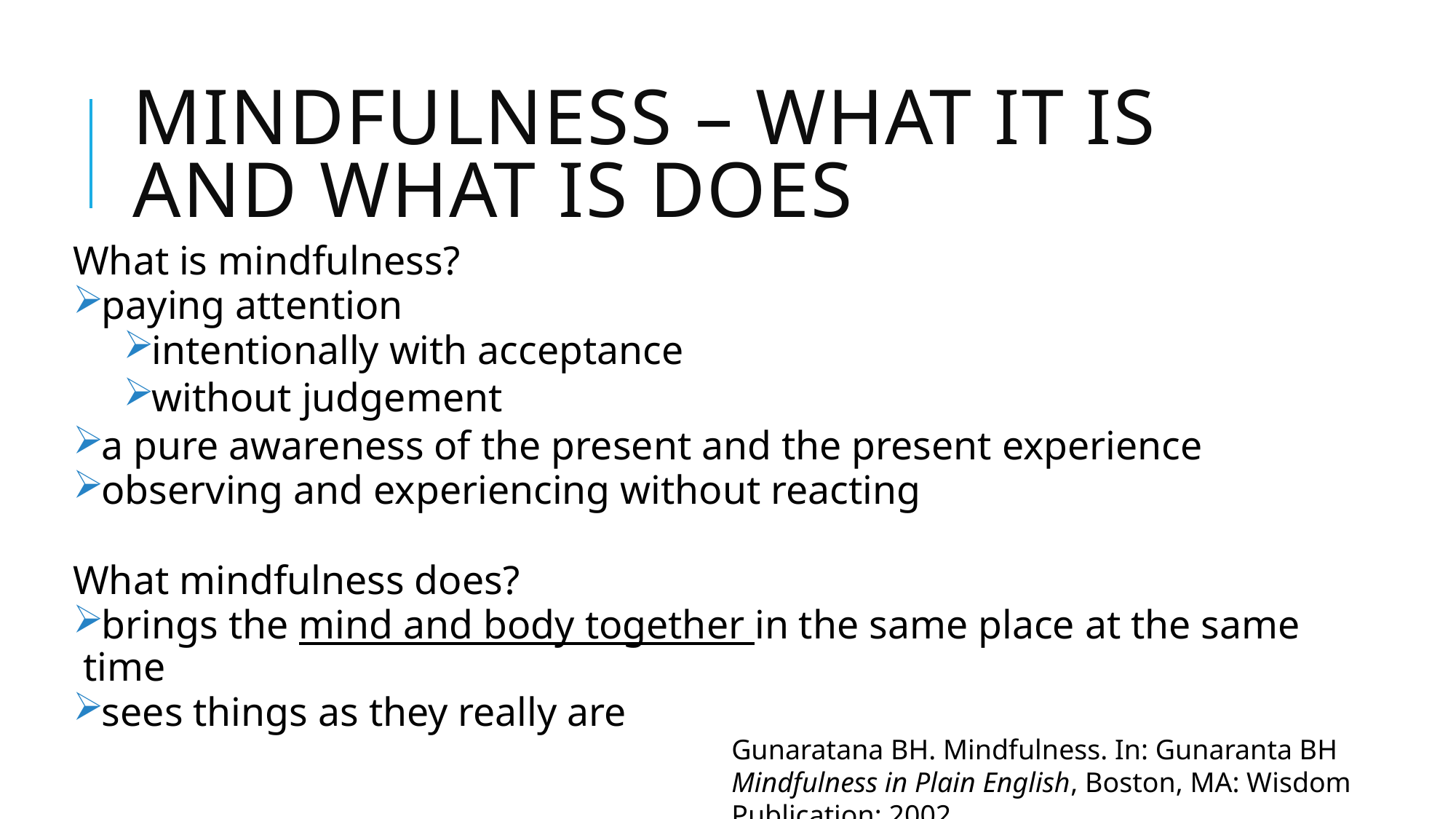

# Mindfulness – what it is and what is does
What is mindfulness?
paying attention
intentionally with acceptance
without judgement
a pure awareness of the present and the present experience
observing and experiencing without reacting
What mindfulness does?
brings the mind and body together in the same place at the same time
sees things as they really are
Gunaratana BH. Mindfulness. In: Gunaranta BH Mindfulness in Plain English, Boston, MA: Wisdom Publication; 2002

## Slide 5
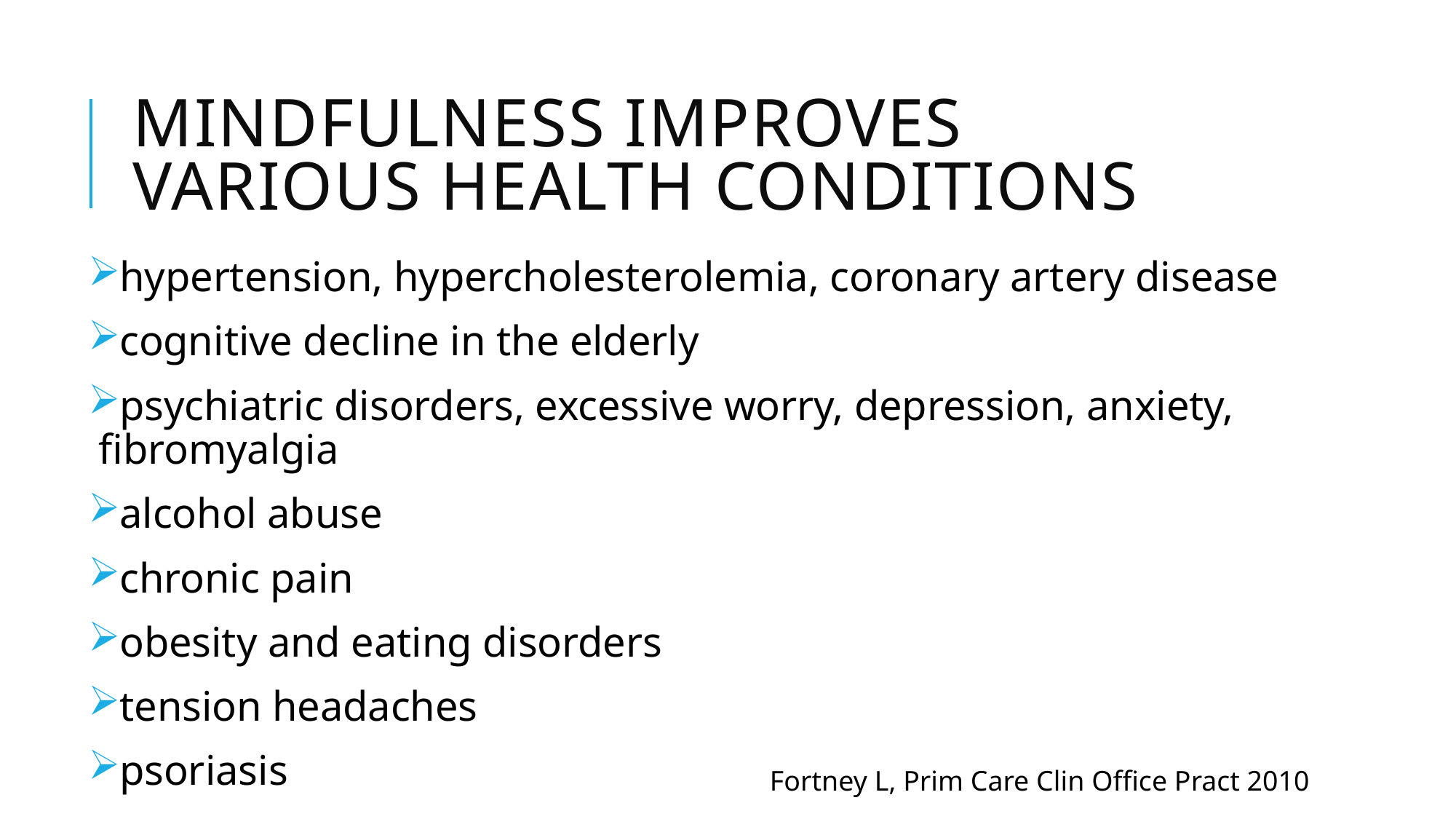

# Mindfulness improves various health conditions
hypertension, hypercholesterolemia, coronary artery disease
cognitive decline in the elderly
psychiatric disorders, excessive worry, depression, anxiety, fibromyalgia
alcohol abuse
chronic pain
obesity and eating disorders
tension headaches
psoriasis
Fortney L, Prim Care Clin Office Pract 2010

## Slide 6
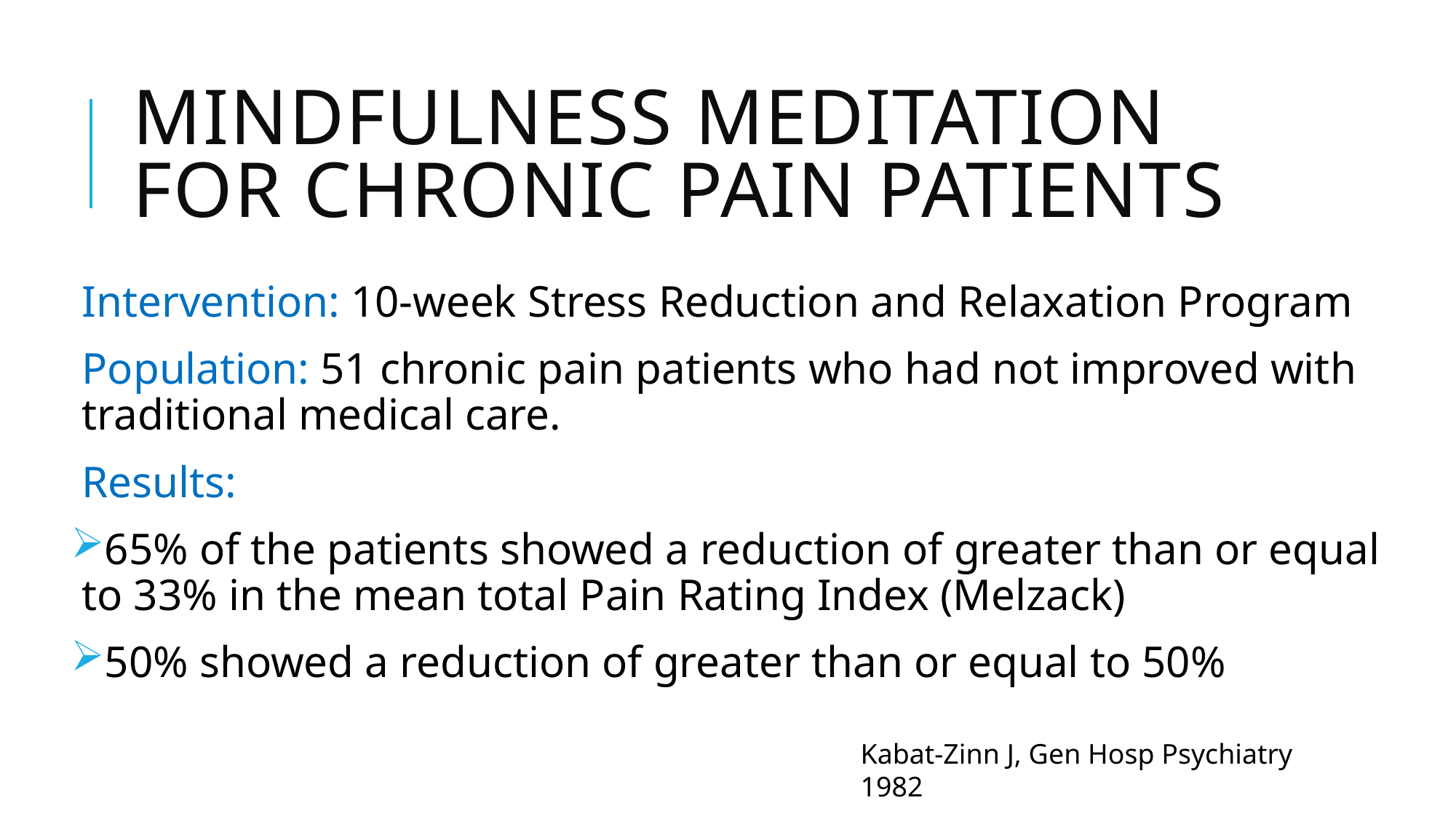

# Mindfulness meditation for chronic pain patients
Intervention: 10-week Stress Reduction and Relaxation Program
Population: 51 chronic pain patients who had not improved with traditional medical care.
Results:
65% of the patients showed a reduction of greater than or equal to 33% in the mean total Pain Rating Index (Melzack)
50% showed a reduction of greater than or equal to 50%
Kabat-Zinn J, Gen Hosp Psychiatry 1982

## Slide 7
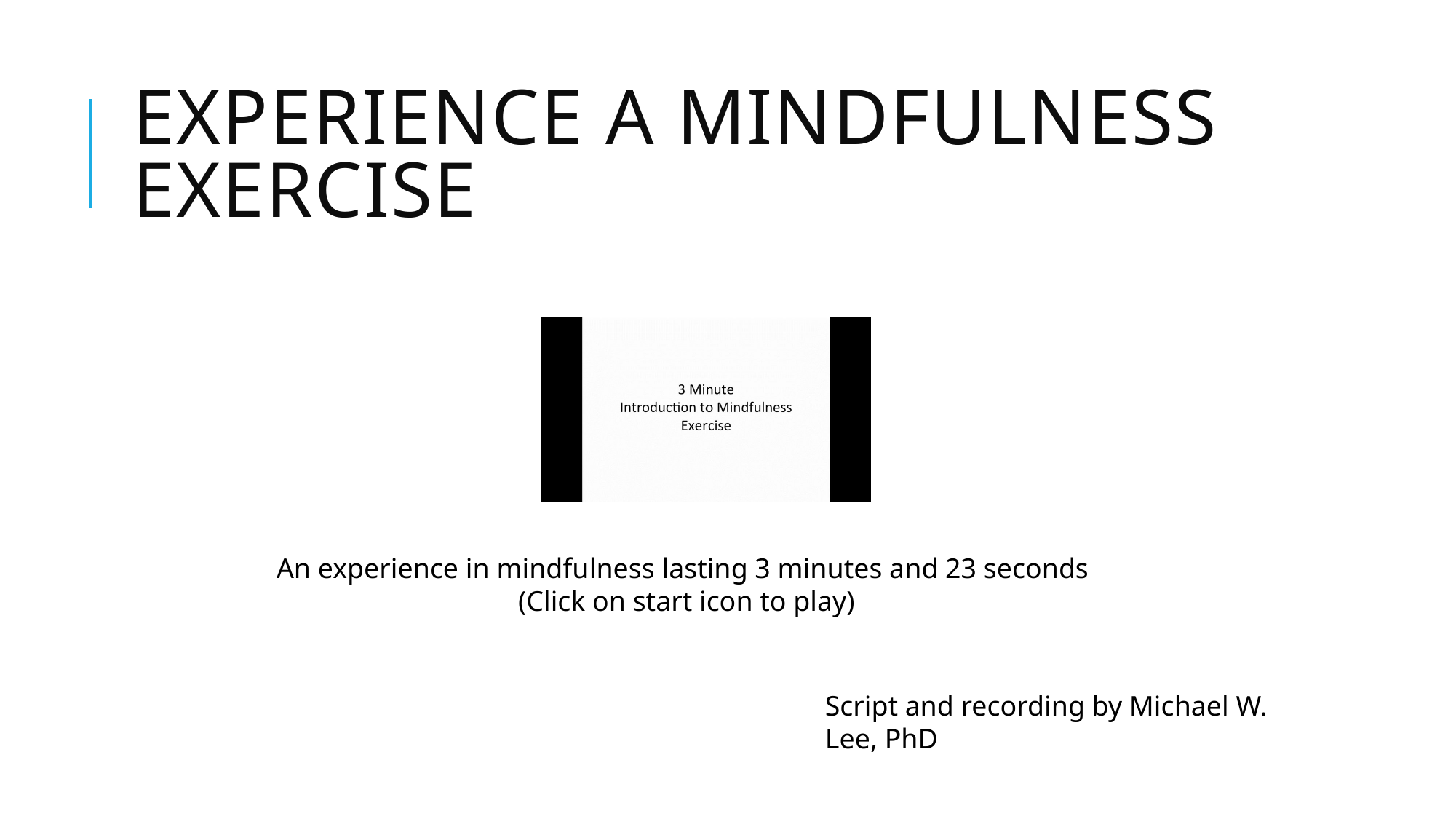

# Experience A Mindfulness Exercise
An experience in mindfulness lasting 3 minutes and 23 seconds
(Click on start icon to play)
Script and recording by Michael W. Lee, PhD

## Slide 8
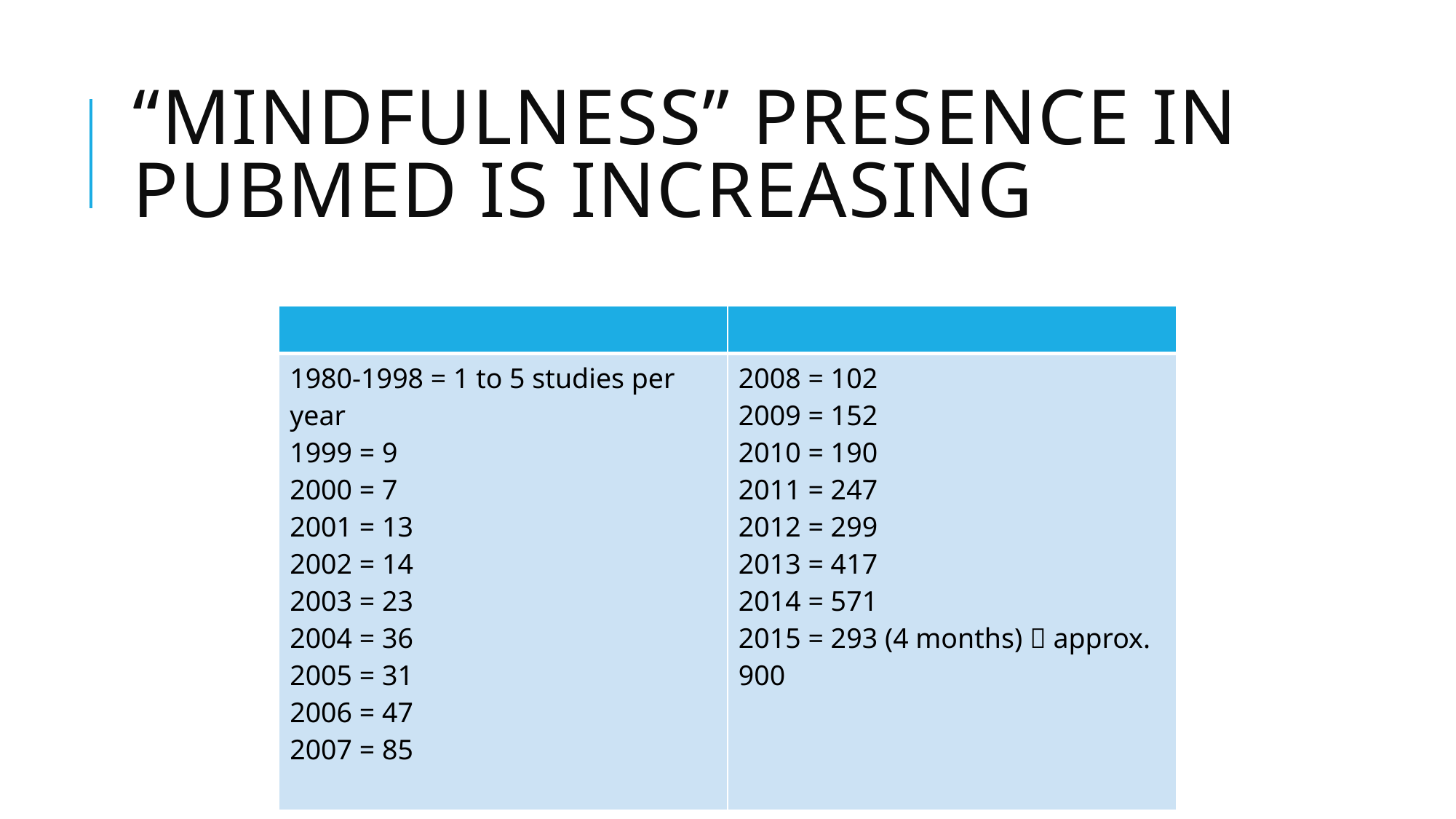

# “MINDFULNESS” presence in PubMed is increasing
| | |
| --- | --- |
| 1980-1998 = 1 to 5 studies per year 1999 = 9 2000 = 7 2001 = 13 2002 = 14 2003 = 23 2004 = 36 2005 = 31 2006 = 47 2007 = 85 | 2008 = 102 2009 = 152 2010 = 190 2011 = 247 2012 = 299 2013 = 417 2014 = 571 2015 = 293 (4 months)  approx. 900 |

## Slide 9
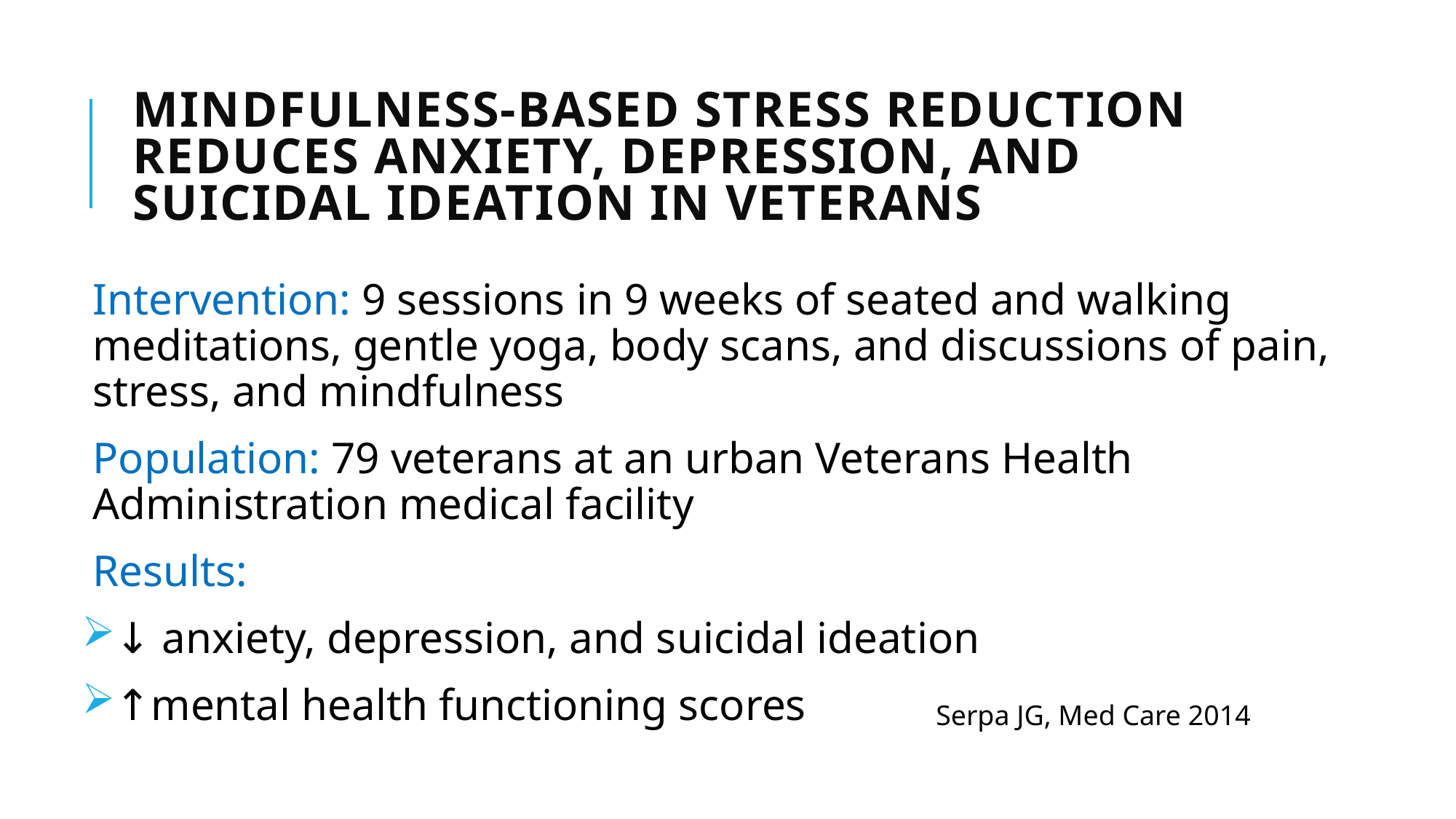

# Mindfulness-based stress reduction reduces anxiety, depression, and suicidal ideation in veterans
Intervention: 9 sessions in 9 weeks of seated and walking meditations, gentle yoga, body scans, and discussions of pain, stress, and mindfulness
Population: 79 veterans at an urban Veterans Health Administration medical facility
Results:
↓ anxiety, depression, and suicidal ideation
↑mental health functioning scores
Serpa JG, Med Care 2014

## Slide 10
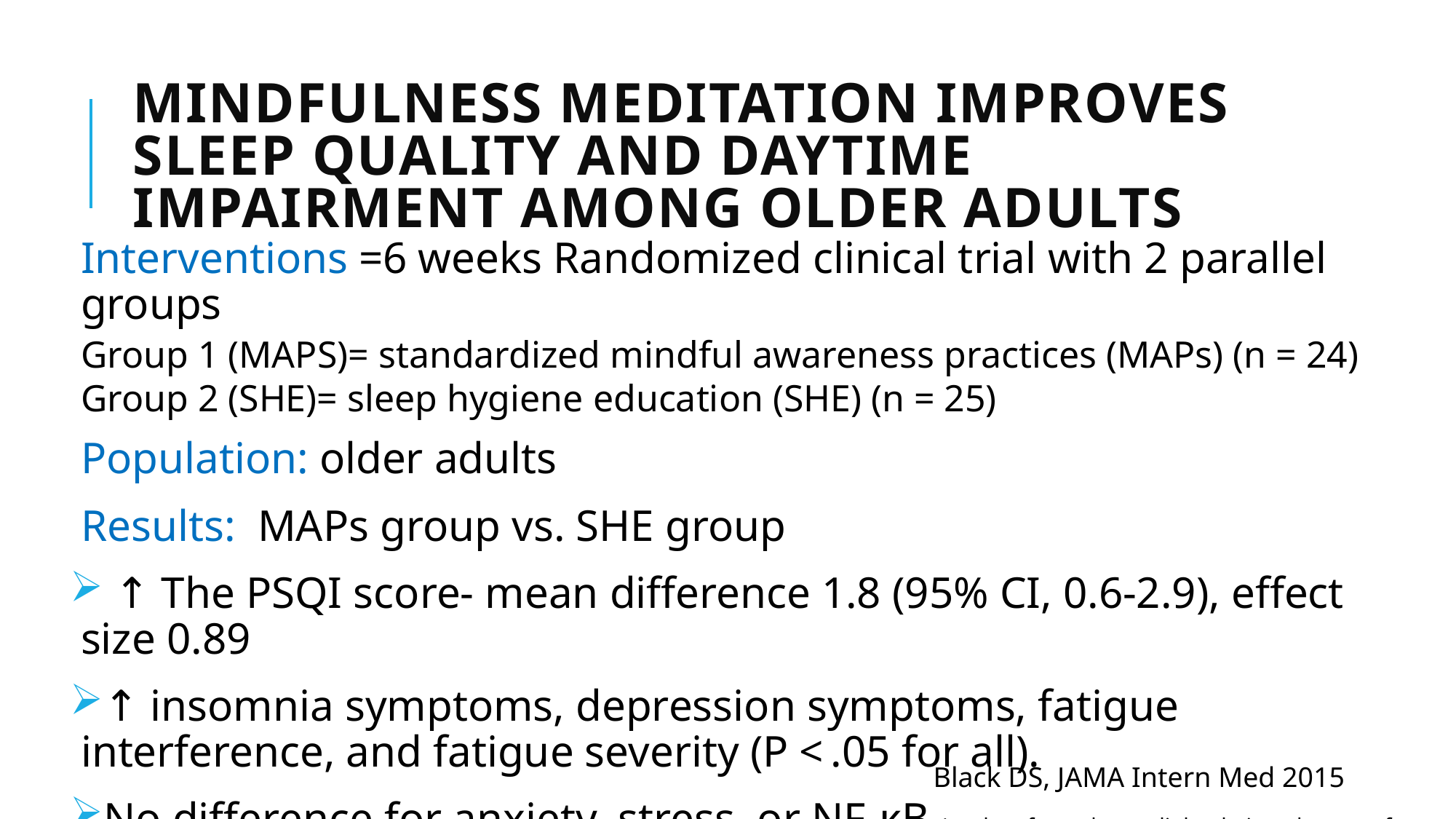

# Mindfulness meditation improves sleep quality and daytime impairment among older adults
Interventions =6 weeks Randomized clinical trial with 2 parallel groups
Group 1 (MAPS)= standardized mindful awareness practices (MAPs) (n = 24)
Group 2 (SHE)= sleep hygiene education (SHE) (n = 25)
Population: older adults
Results: MAPs group vs. SHE group
 ↑ The PSQI score- mean difference 1.8 (95% CI, 0.6-2.9), effect size 0.89
↑ insomnia symptoms, depression symptoms, fatigue interference, and fatigue severity (P < .05 for all).
No difference for anxiety, stress, or NF-κB (nuclear factor kappa-light-chain-enhancer of activated B cells)
Black DS, JAMA Intern Med 2015

## Slide 11
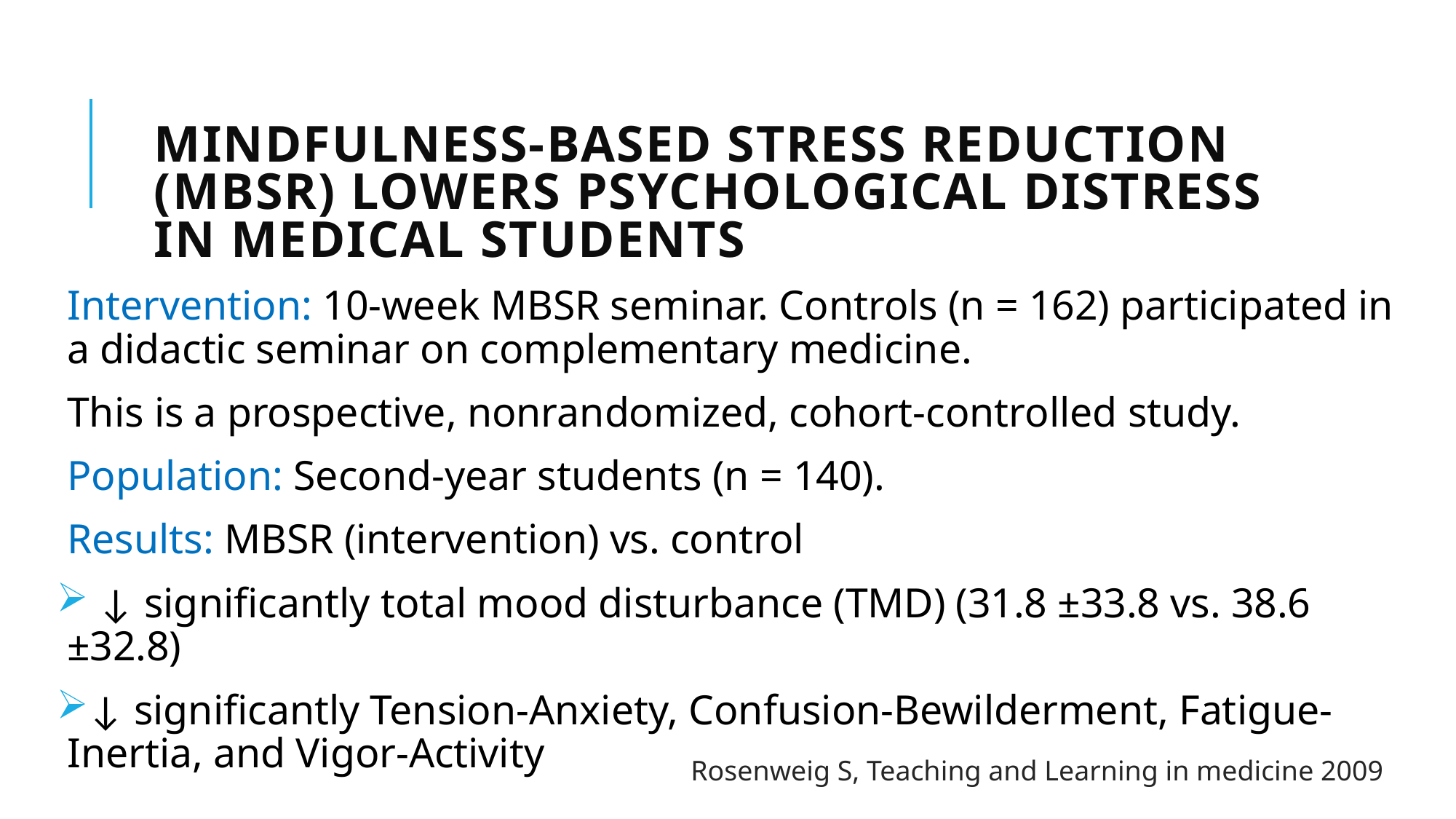

# Mindfulness-Based Stress Reduction (MBSR) Lowers Psychological Distress In Medical Students
Intervention: 10-week MBSR seminar. Controls (n = 162) participated in a didactic seminar on complementary medicine.
This is a prospective, nonrandomized, cohort-controlled study.
Population: Second-year students (n = 140).
Results: MBSR (intervention) vs. control
 ↓ significantly total mood disturbance (TMD) (31.8 ±33.8 vs. 38.6 ±32.8)
↓ significantly Tension-Anxiety, Confusion-Bewilderment, Fatigue-Inertia, and Vigor-Activity
Rosenweig S, Teaching and Learning in medicine 2009

## Slide 12
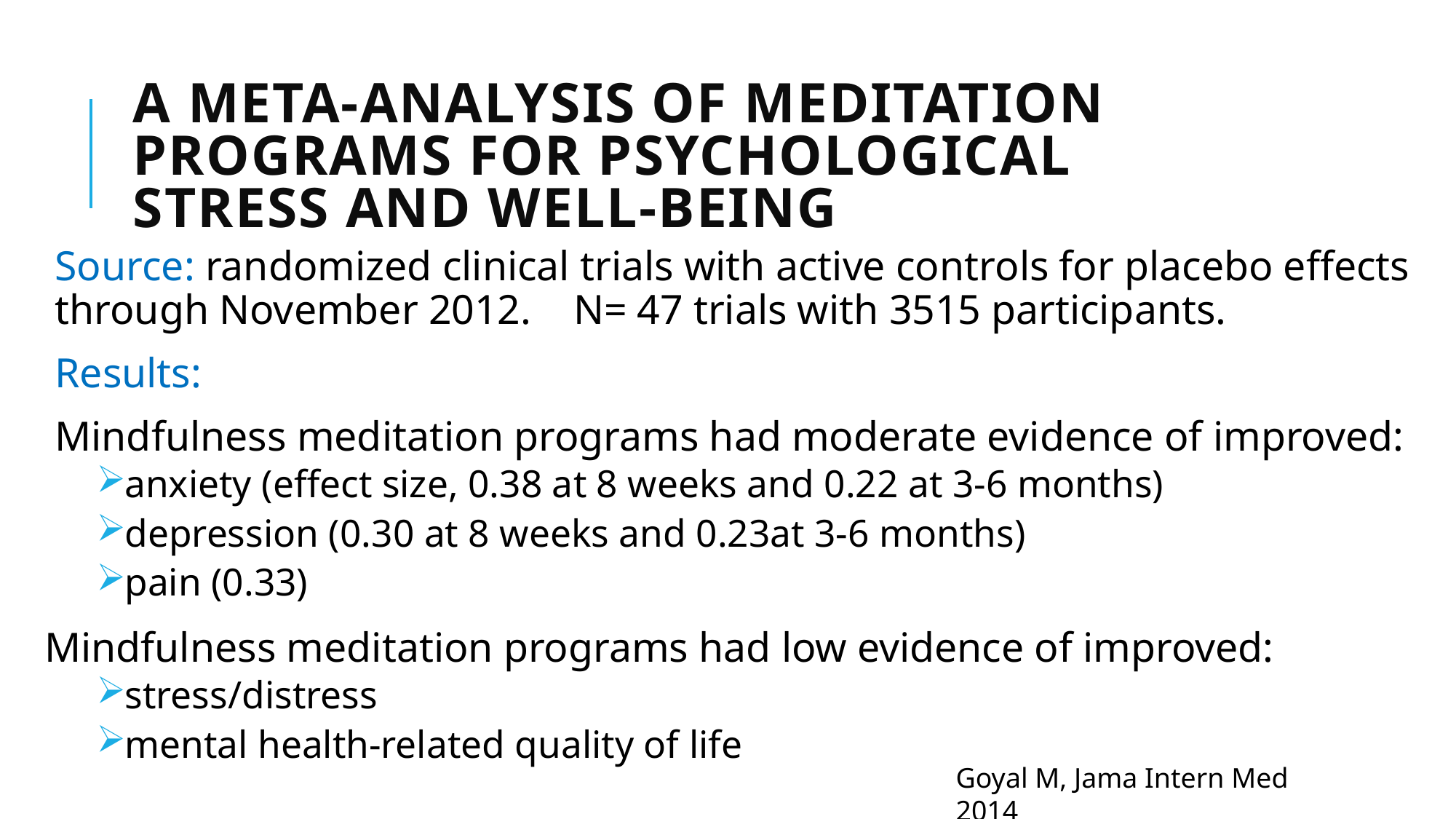

# A meta-analysis of meditation programs for psychological stress and well-being
Source: randomized clinical trials with active controls for placebo effects through November 2012. 		N= 47 trials with 3515 participants.
Results:
Mindfulness meditation programs had moderate evidence of improved:
anxiety (effect size, 0.38 at 8 weeks and 0.22 at 3-6 months)
depression (0.30 at 8 weeks and 0.23at 3-6 months)
pain (0.33)
Mindfulness meditation programs had low evidence of improved:
stress/distress
mental health-related quality of life
Goyal M, Jama Intern Med 2014

## Slide 13
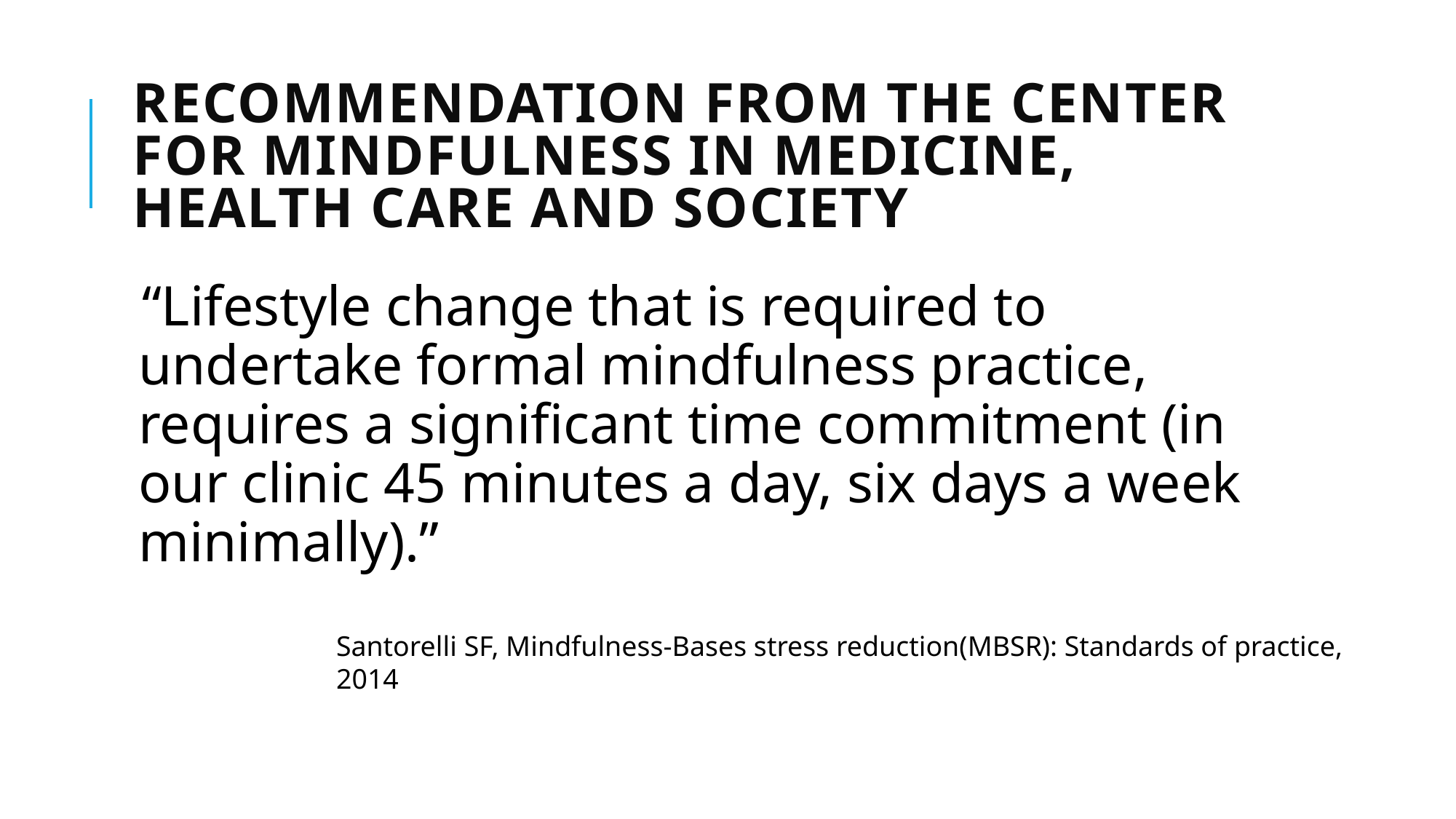

# Recommendation from the Center for Mindfulness in medicine, Health Care and Society
“Lifestyle change that is required to undertake formal mindfulness practice, requires a significant time commitment (in our clinic 45 minutes a day, six days a week minimally).”
Santorelli SF, Mindfulness-Bases stress reduction(MBSR): Standards of practice, 2014

## Slide 14
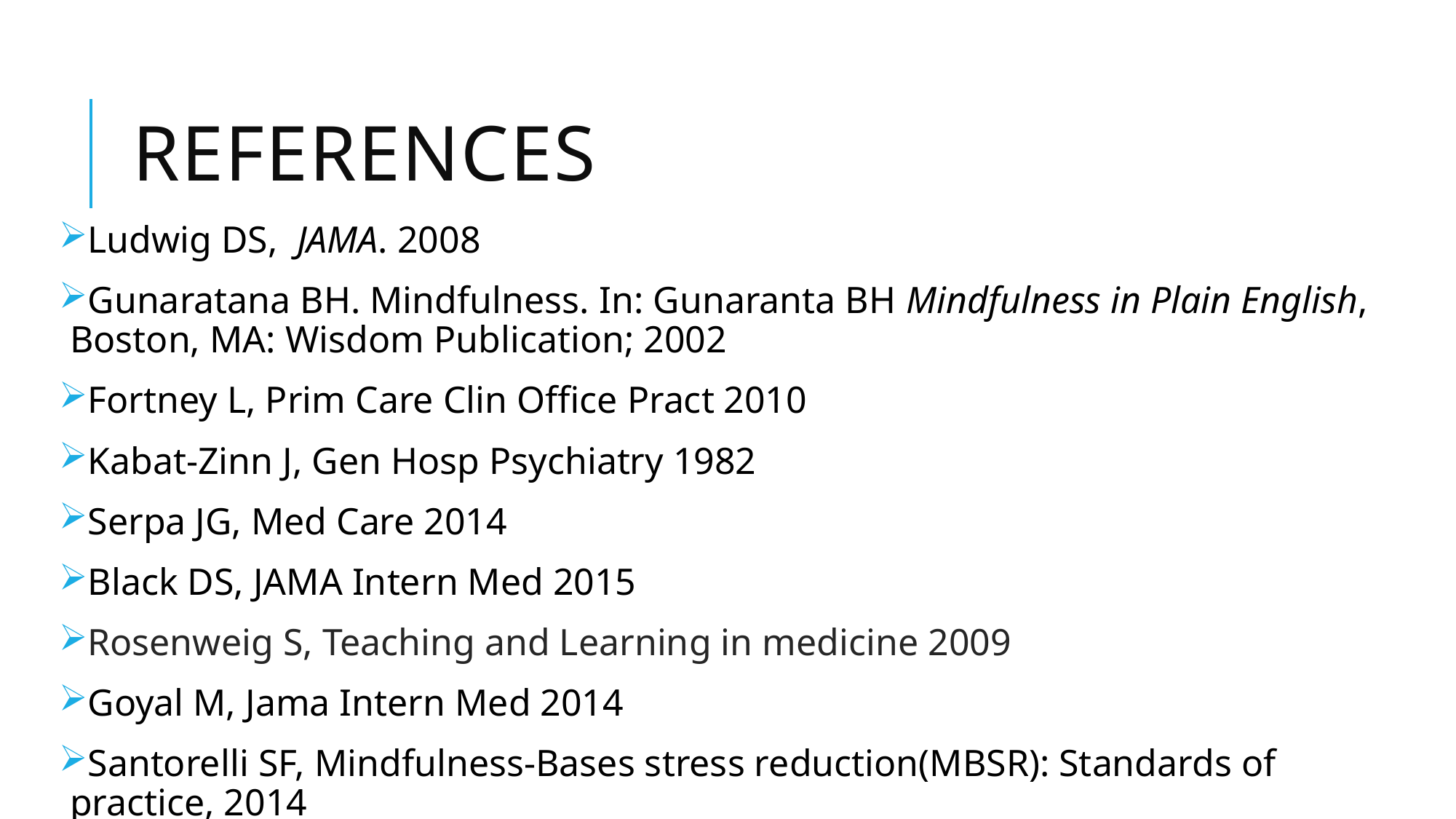

# References
Ludwig DS, JAMA. 2008
Gunaratana BH. Mindfulness. In: Gunaranta BH Mindfulness in Plain English, Boston, MA: Wisdom Publication; 2002
Fortney L, Prim Care Clin Office Pract 2010
Kabat-Zinn J, Gen Hosp Psychiatry 1982
Serpa JG, Med Care 2014
Black DS, JAMA Intern Med 2015
Rosenweig S, Teaching and Learning in medicine 2009
Goyal M, Jama Intern Med 2014
Santorelli SF, Mindfulness-Bases stress reduction(MBSR): Standards of practice, 2014

## Slide 15
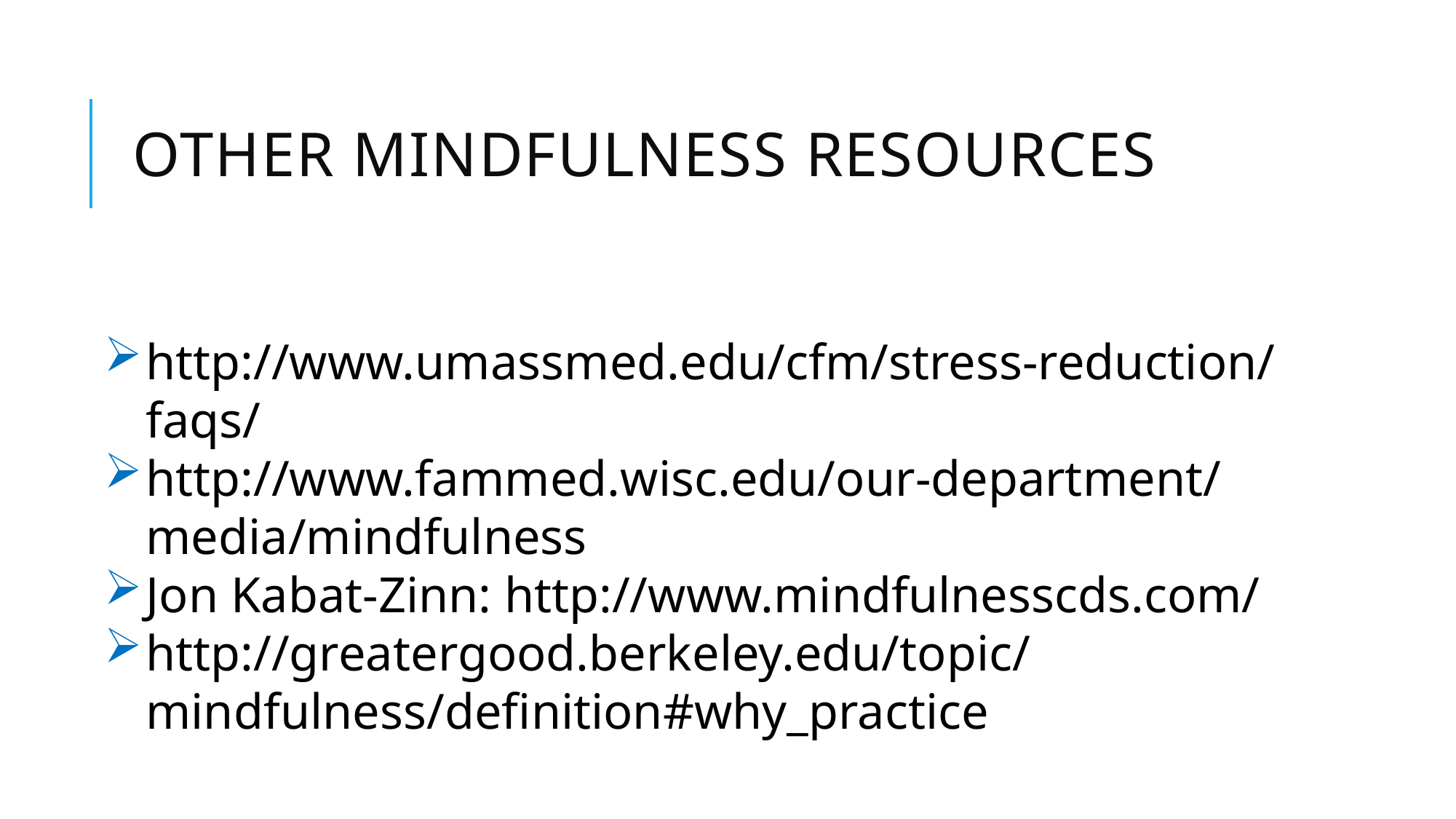

# Other Mindfulness Resources
http://www.umassmed.edu/cfm/stress-reduction/faqs/
http://www.fammed.wisc.edu/our-department/media/mindfulness
Jon Kabat-Zinn: http://www.mindfulnesscds.com/
http://greatergood.berkeley.edu/topic/mindfulness/definition#why_practice
