# Supplementary material for: Introduction to Mindfulness: Evidence-Based Medicine Lecture and Active Session
Source: MedEdPORTAL. 2016 Sep 28;12:10472. doi: 10.15766/mep_2374-8265.10472 (PMC6464426; doi:10.15766/mep_2374-8265.10472)
Supplement: Supplementary file 1 — A. Mindfulness Presentation.pptx B. Survey-Electronic.pptx C. Survey-Paper.docx [file mep-12-10472-s001.zip › B. Survey-Electronic.pptx]

## Slide 1
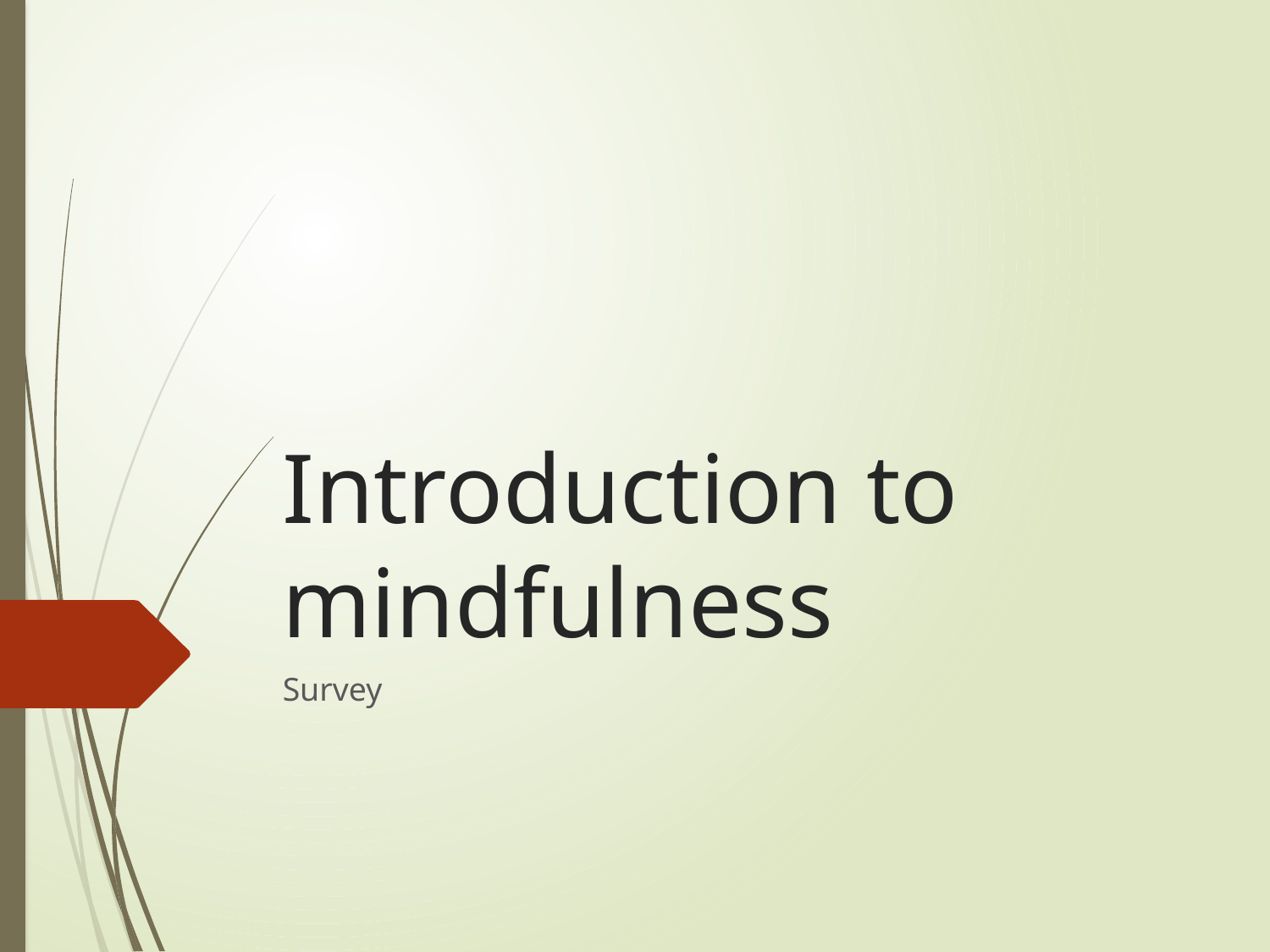

# Introduction to mindfulness
Survey

## Slide 2
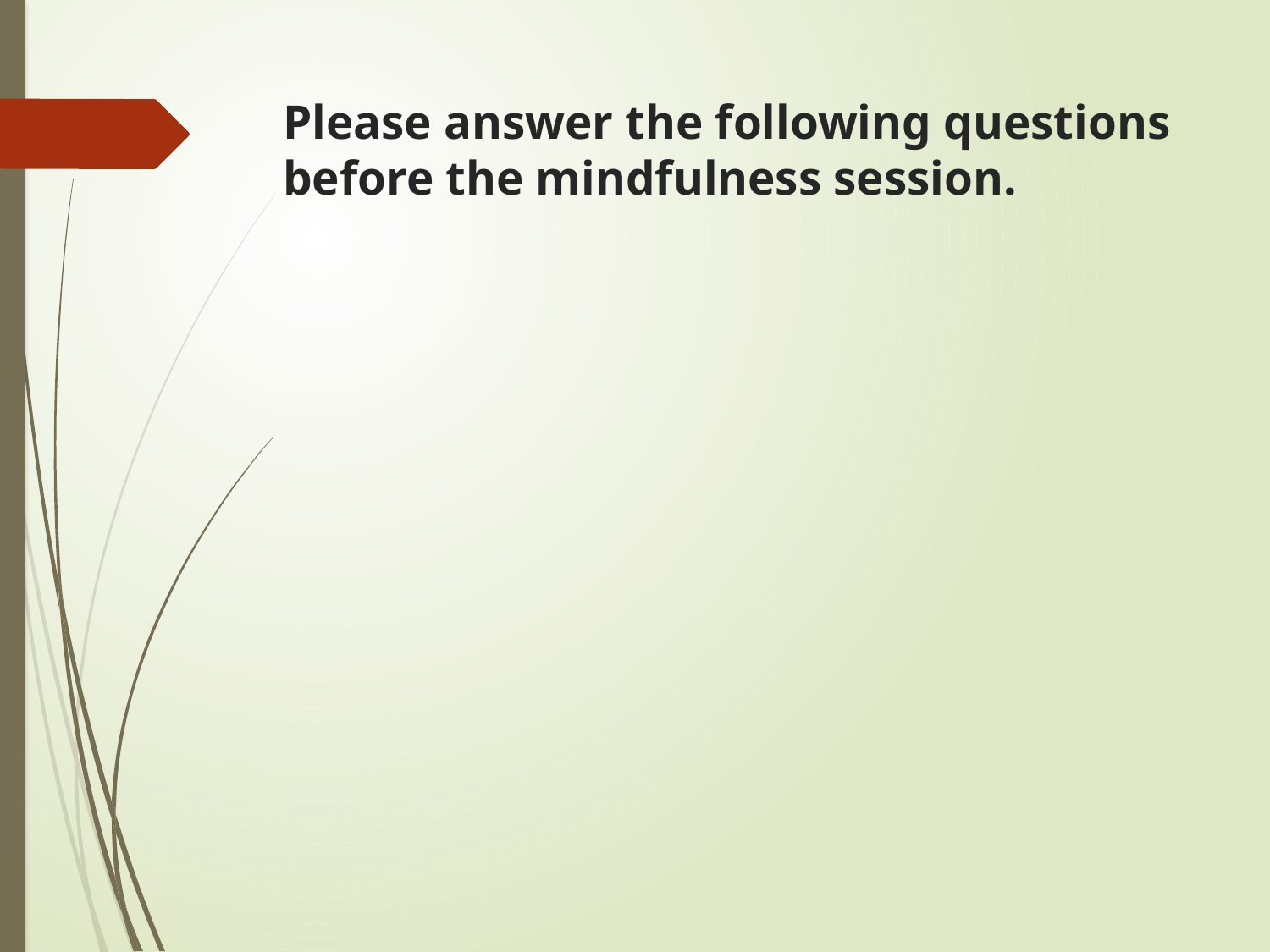

# Please answer the following questions before the mindfulness session.

## Slide 3
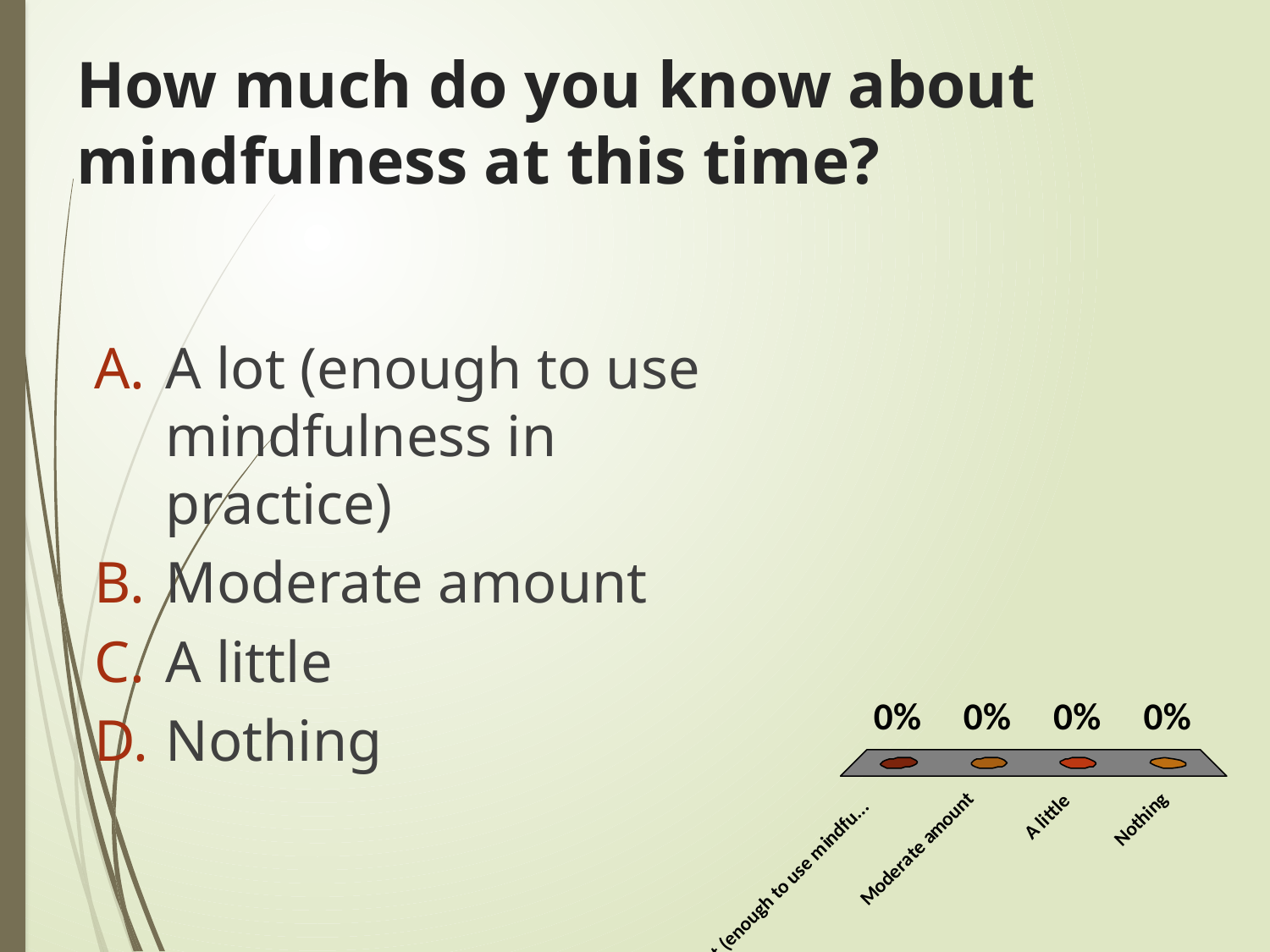

# How much do you know about mindfulness at this time?
A lot (enough to use mindfulness in practice)
Moderate amount
A little
Nothing

## Slide 4
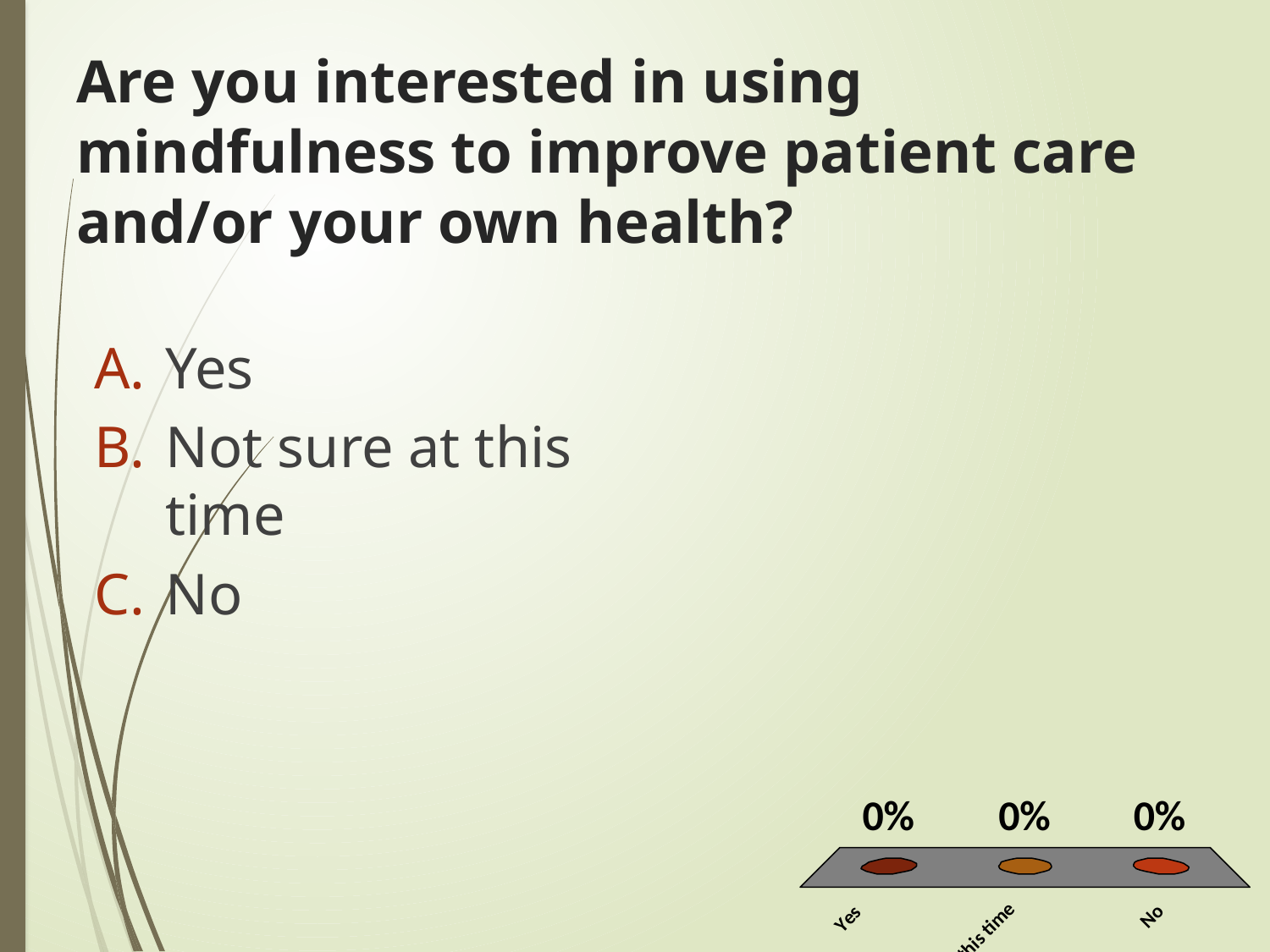

# Are you interested in using mindfulness to improve patient care and/or your own health?
Yes
Not sure at this time
No

## Slide 5
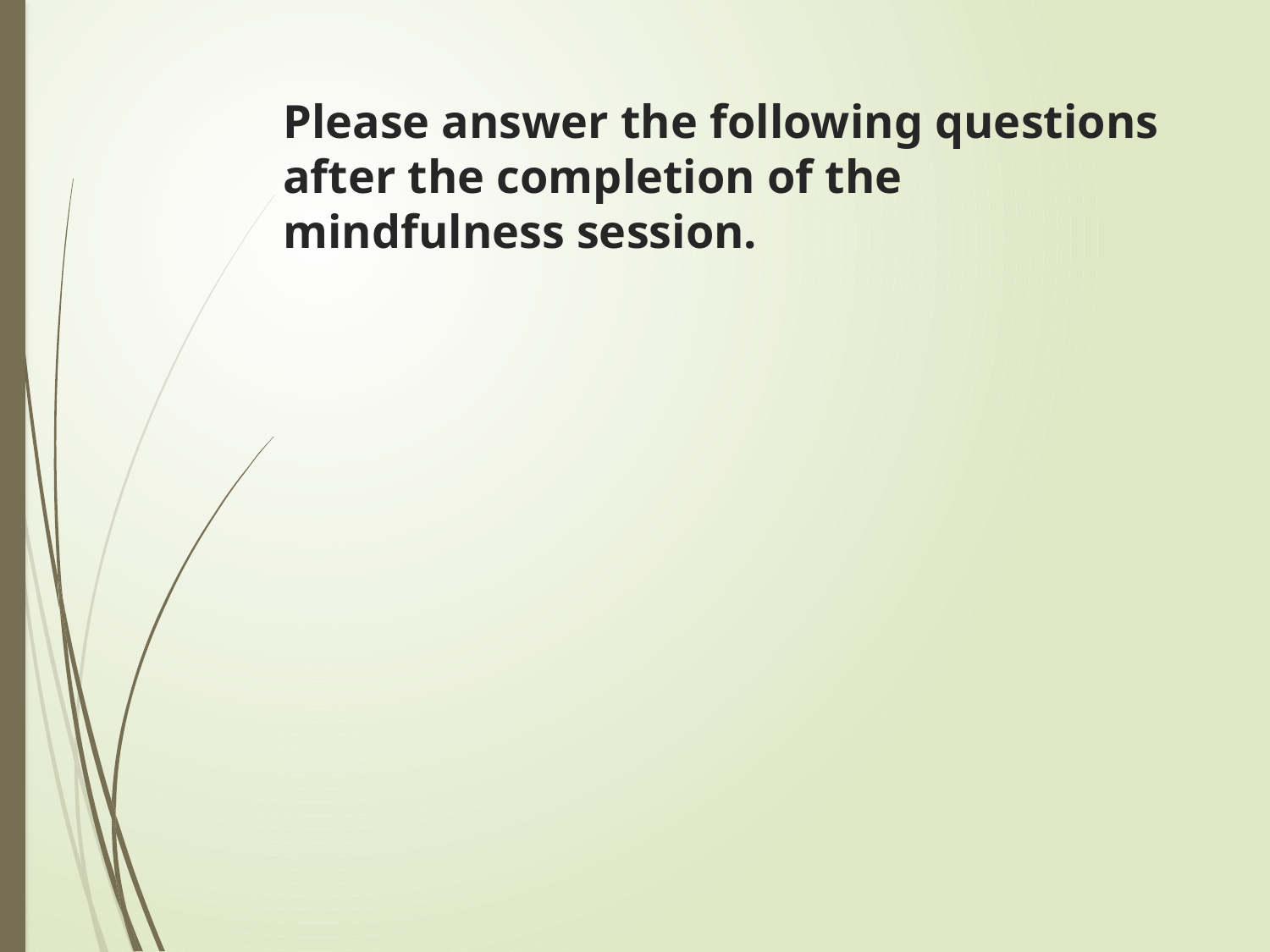

# Please answer the following questions after the completion of the mindfulness session.

## Slide 6
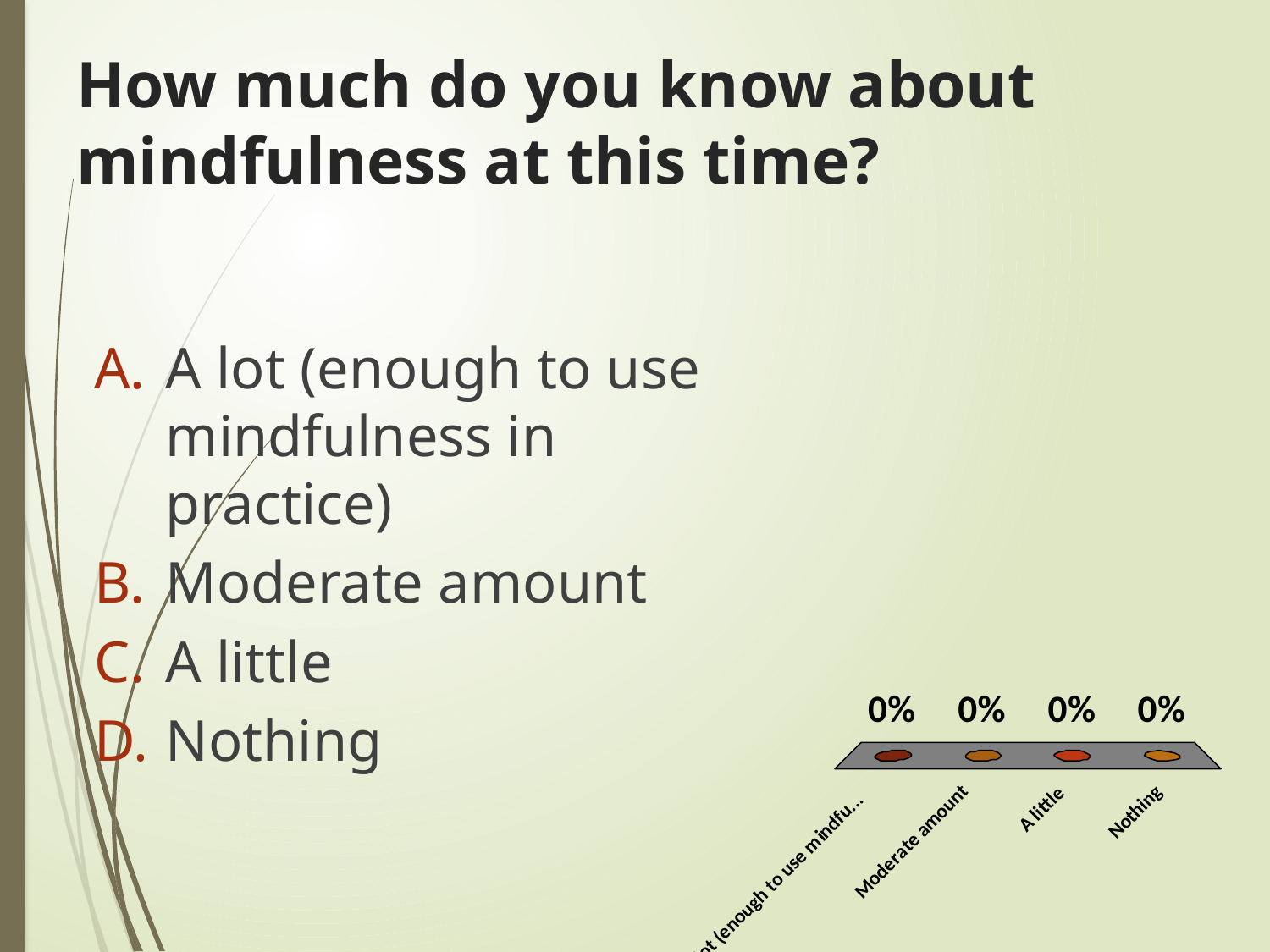

# How much do you know about mindfulness at this time?
A lot (enough to use mindfulness in practice)
Moderate amount
A little
Nothing

## Slide 7
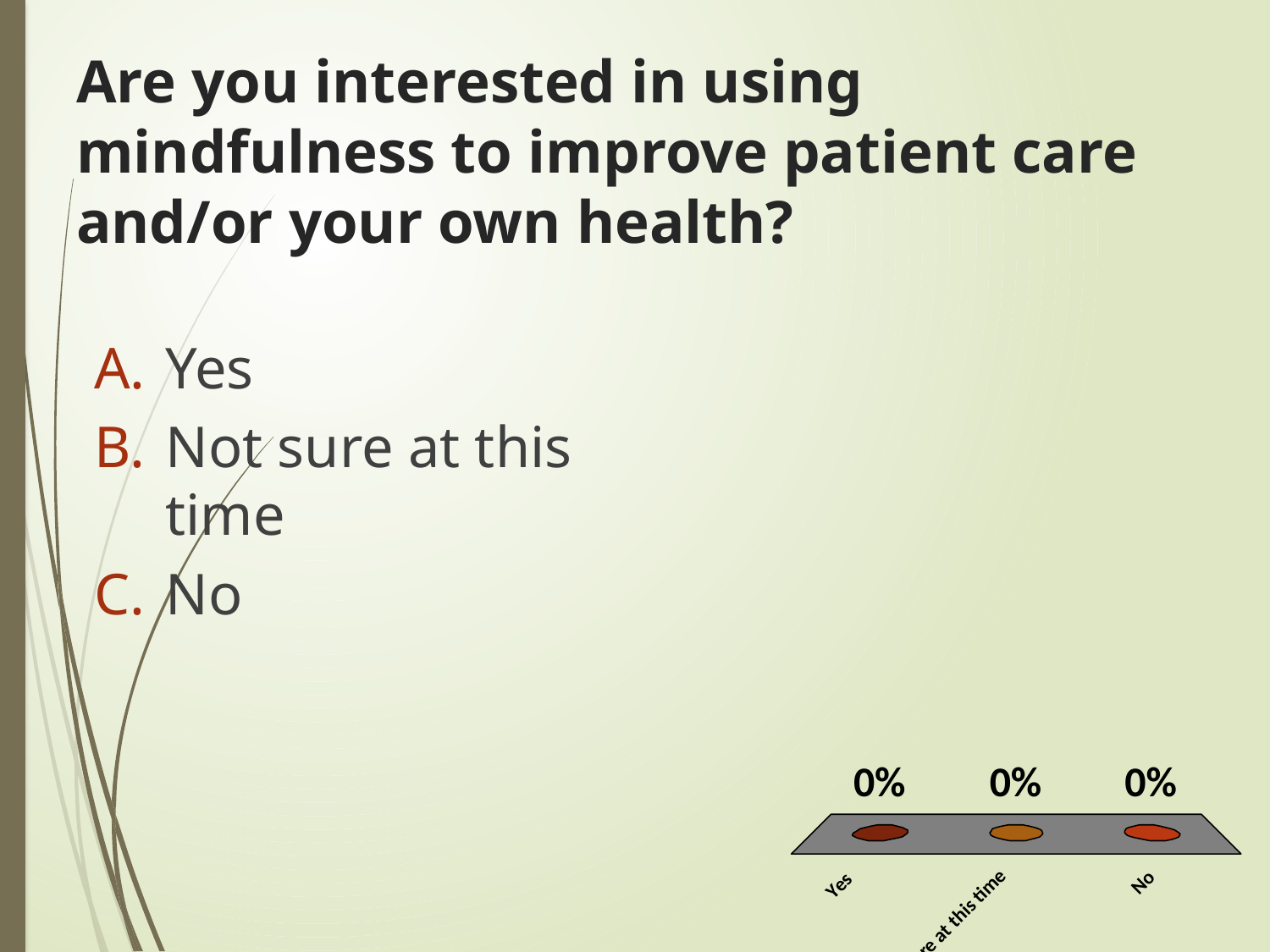

# Are you interested in using mindfulness to improve patient care and/or your own health?
Yes
Not sure at this time
No

## Slide 8
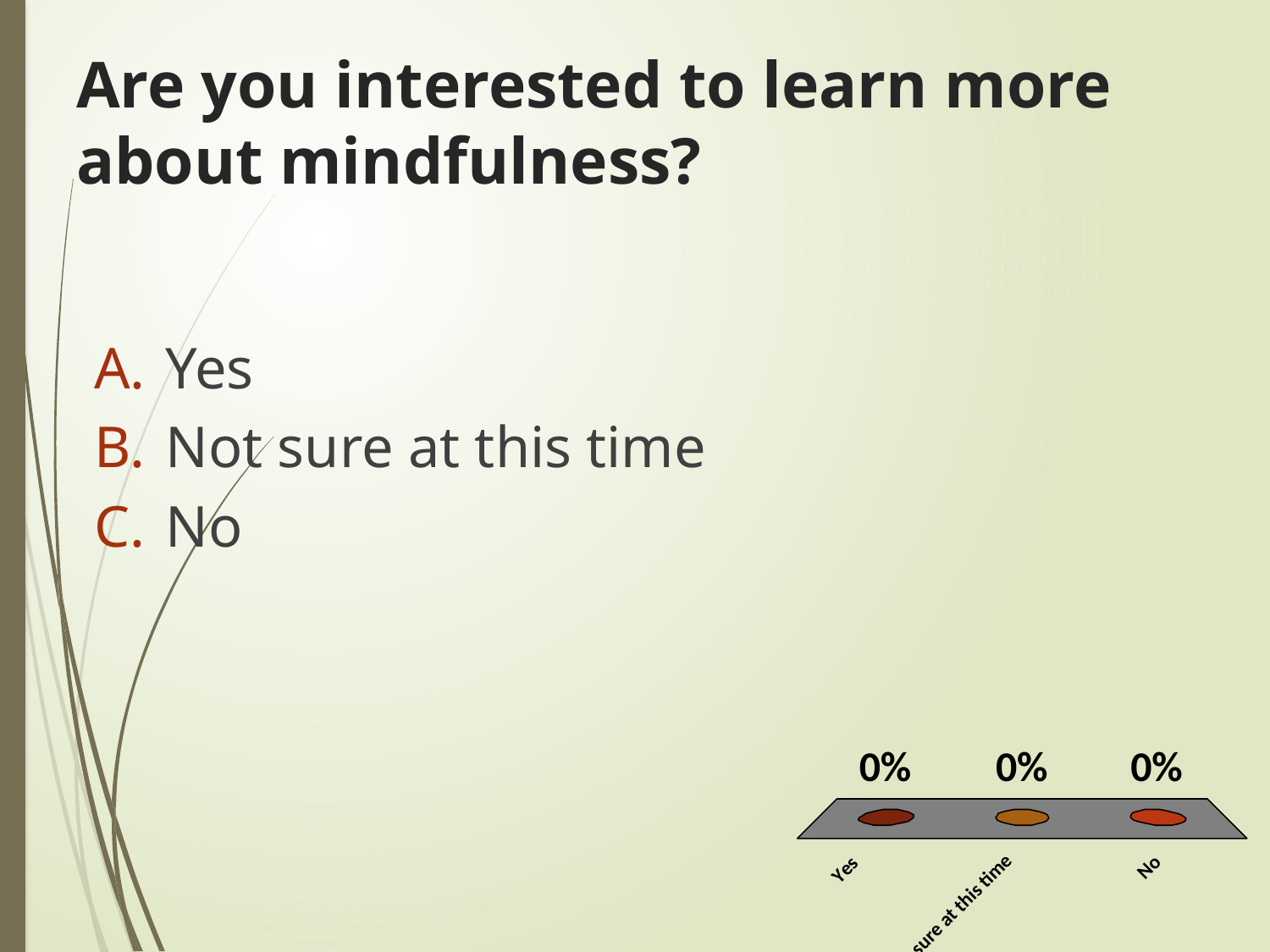

# Are you interested to learn more about mindfulness?
Yes
Not sure at this time
No

## Slide 9
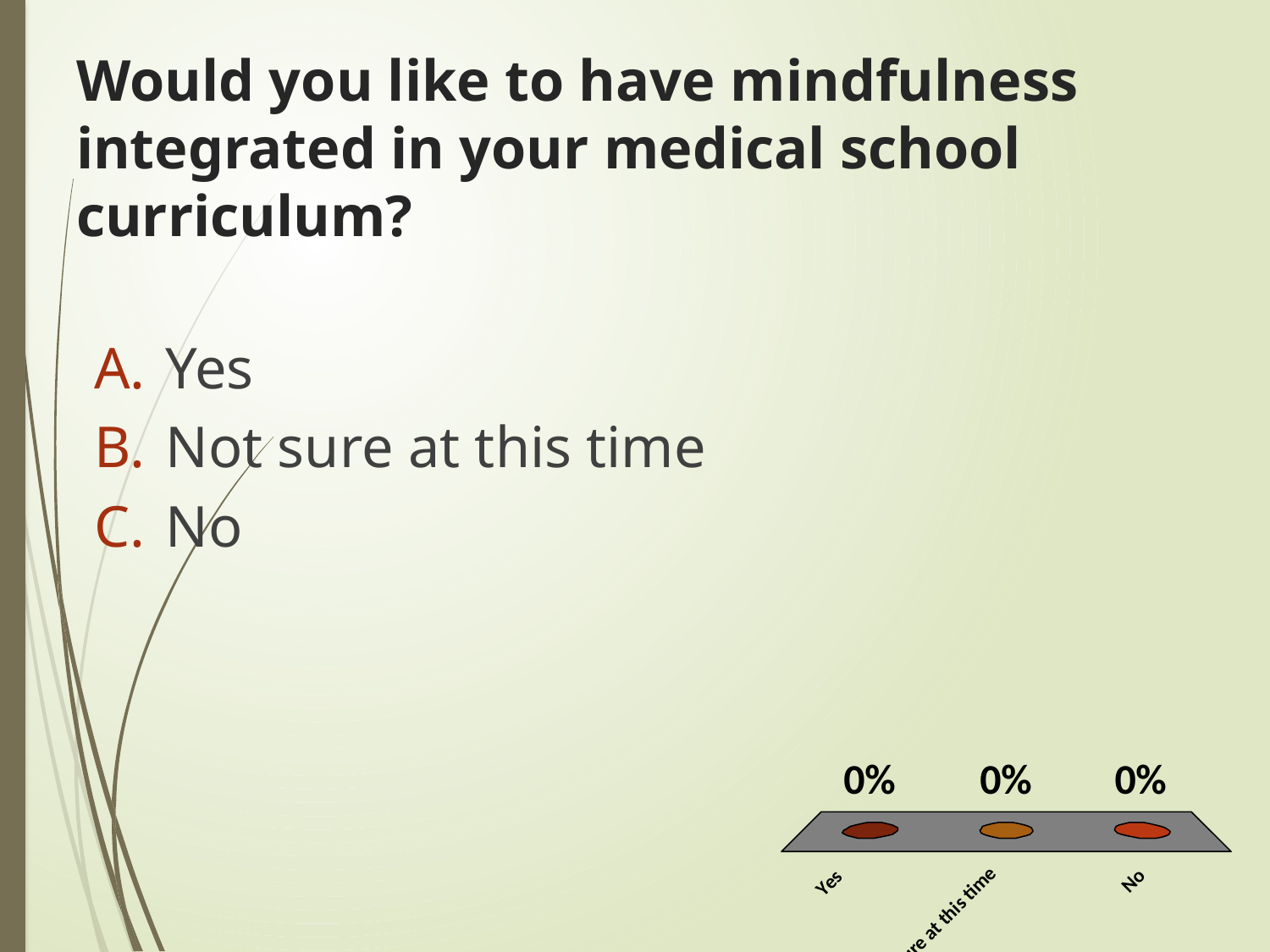

# Would you like to have mindfulness integrated in your medical school curriculum?
Yes
Not sure at this time
No
