# Supplementary material for: Introduction to Mindfulness: Evidence-Based Medicine Lecture and Active Session
Source: MedEdPORTAL. 2016 Sep 28;12:10472. doi: 10.15766/mep_2374-8265.10472 (PMC6464426; doi:10.15766/mep_2374-8265.10472)
Supplement: Supplementary file 1 — A. Mindfulness Presentation.pptx B. Survey-Electronic.pptx C. Survey-Paper.docx [file mep-12-10472-s001.zip › C. Survey-Paper.docx]

**Introduction to mindfulness survey**

**Please answer the following questions before the mindfulness session.**

1. **How much do you know about mindfulness at this time?**
2. A lot (enough to use mindfulness in practice)
3. Moderate amount
4. A little
5. Nothing
6. **Are you interested in using mindfulness to improve patient care and/or your own health?**
7. Yes
8. Not sure at this time
9. No

**Please answer the following questions after the completion of the mindfulness session.**

1. **How much do you know about mindfulness at this time?**
2. A lot (enough to use mindfulness in practice)
3. Moderate amount
4. A little
5. Nothing
6. **Are you interested in using mindfulness to improve patient care and/or your own health?**
7. Yes
8. Not sure at this time
9. No
10. **Are you interested to learn more about mindfulness?**
11. Yes
12. Not sure at this time
13. No
14. **Would you like to have mindfulness integrated in your medical school curriculum?**
15. Yes
16. Not sure at this time
17. No
